# Supplementary material for: Pb-resistant Pantoea rwandensis promotes maize’s growth by altering Pb accumulation in biomass and soil Pb immobilization
Source: PLoS One. 2024 Oct 18;19(10):e0306392. doi: 10.1371/journal.pone.0306392 (PMC11488736; doi:10.1371/journal.pone.0306392)
Supplement: S1 Fig — (A) The change in the dissolved P content and the pH value in the broth medium; (B) the OD values at 600 nm after culturing the PSB in fermentation liquid with Pb2+ concentrations of 0, 250, 500, 750, or 1000 mg·L-1 for 72 h; (C) the content of organic acid and IAA secreted by the PSB; (D) the phylogenetic tree of J101 based on the 16S rDNA gene sequences; and (E) the root systems of maize under different treatments. (DOCX) [file pone.0306392.s001.docx]

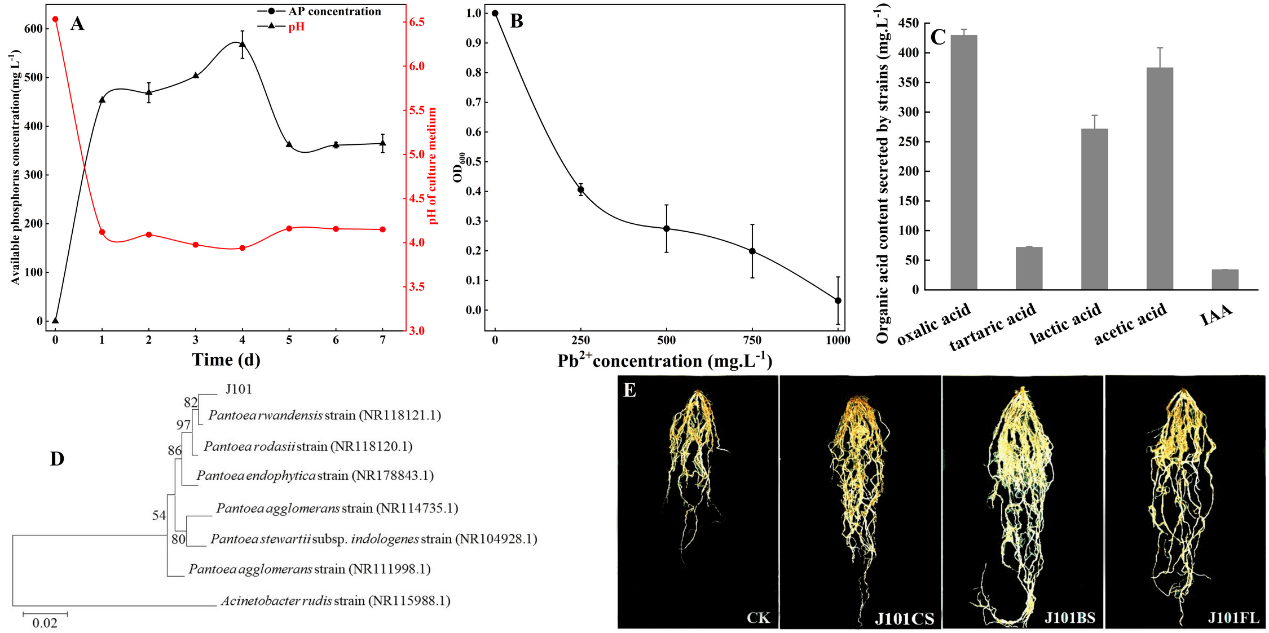


**S1 Fig. Analysis of dissolved P, Pb tolerance, and secreted metabolites of J101.** (A) The change in the dissolved P content and the pH value in the broth medium; (B) the OD values at 600 nm after culturing the PSB in fermentation liquid with Pb^2+^ concentrations of 0, 250, 500, 750, or 1000 mg·L^-1^ for 72 h; (C) the content of organic acid and IAA secreted by the PSB; (D) the phylogenetic tree of J101 based on the 16S rDNA gene sequences; and (E) the root systems of maize under different treatments.
