# Supplementary material for: Pb-resistant Pantoea rwandensis promotes maize’s growth by altering Pb accumulation in biomass and soil Pb immobilization
Source: PLoS One. 2024 Oct 18;19(10):e0306392. doi: 10.1371/journal.pone.0306392 (PMC11488736; doi:10.1371/journal.pone.0306392)
Supplement: S2 Fig — (A) Pb absorption by maize; (B) changes in superoxide dismutase (SOD) activity in maize; (C) changes in peroxidase (POD) activity in maize; and (D) changes in malondialdehyde (MDA) content in maize. Note: Different lowercase letters indicate significant differences between groups. (DOCX) [file pone.0306392.s002.docx]

**
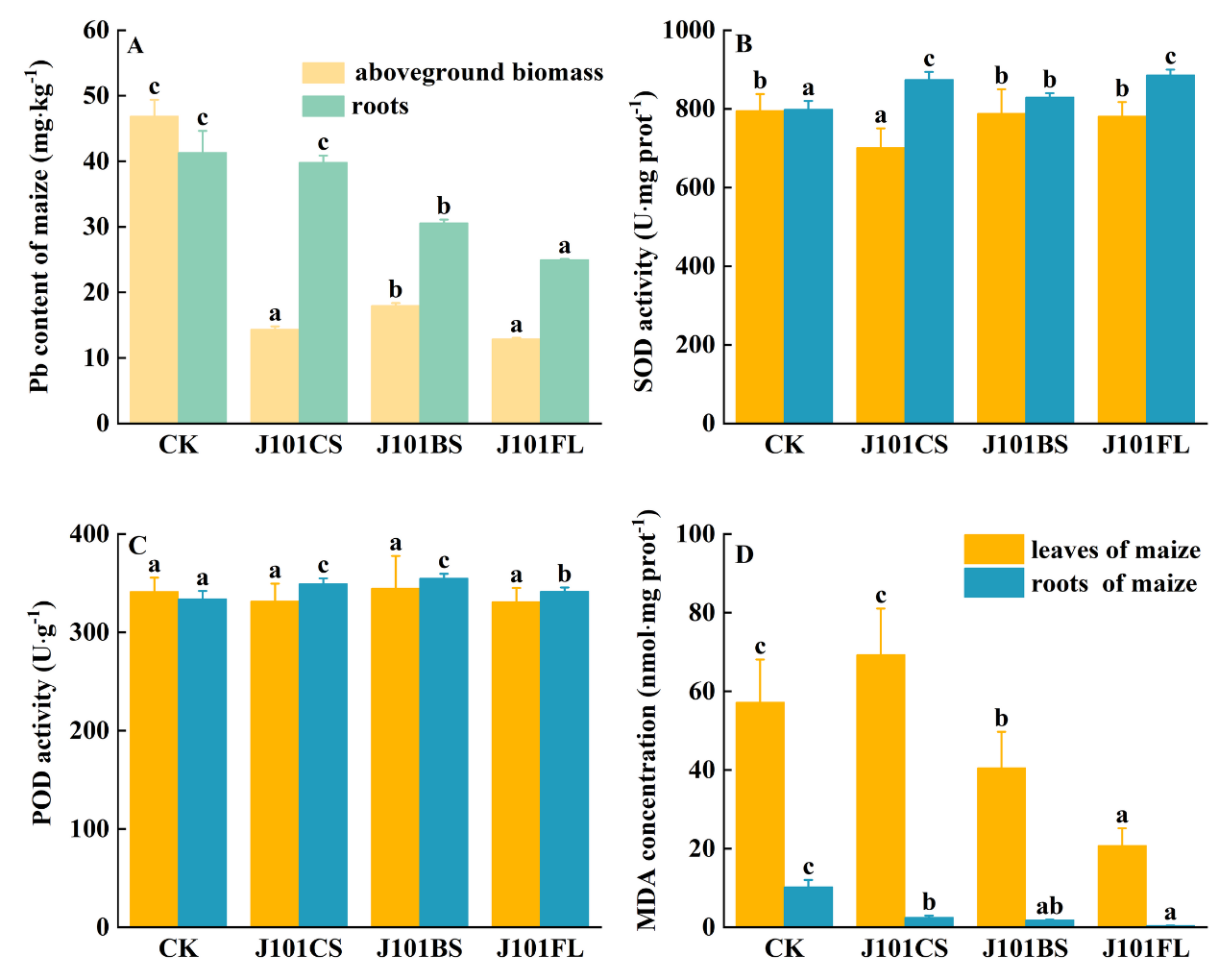
**

**S2 Fig. The effects of different treatments on Pb uptake, antioxidant enzyme activity, and malondialdehyde content in maize.** (A) Pb absorption by maize; (B) changes in superoxide dismutase (SOD) activity in maize; (C) changes in peroxidase (POD) activity in maize; and (D) changes in malondialdehyde (MDA) content in maize. **Note:** Different lowercase letters indicate significant differences between groups.
