# Supplementary material for: Pb-resistant Pantoea rwandensis promotes maize’s growth by altering Pb accumulation in biomass and soil Pb immobilization
Source: PLoS One. 2024 Oct 18;19(10):e0306392. doi: 10.1371/journal.pone.0306392 (PMC11488736; doi:10.1371/journal.pone.0306392)
Supplement: S3 Fig — (A) N uptake by maize; (B) P uptake by maize. Note: Different lowercase letters indicate significant differences between groups. (DOCX) [file pone.0306392.s003.docx]

**
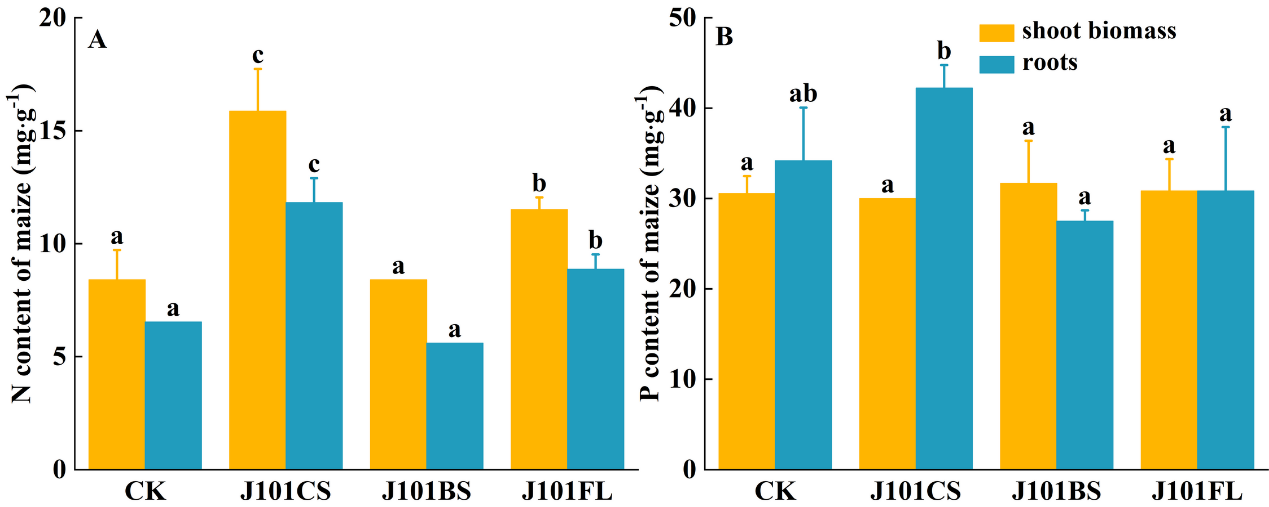
**

**S3 Fig. Changes in the uptake of the nutrient elements N and P by maize. (A) N uptake by maize; (B) P uptake by maize. Note:** Different lowercase letters indicate significant differences between groups.
