# Supplementary material for: Pb-resistant Pantoea rwandensis promotes maize’s growth by altering Pb accumulation in biomass and soil Pb immobilization
Source: PLoS One. 2024 Oct 18;19(10):e0306392. doi: 10.1371/journal.pone.0306392 (PMC11488736; doi:10.1371/journal.pone.0306392)
Supplement: S4 Fig — (A) Changes in the relative abundance of bacteria at the phylum level; (B) changes in the relative abundance of bacteria at the genius level; (C) changes in the relative abundance of fungi at the phylum level; and (D) changes in the relative abundance of fungi at the genius level. (DOCX) [file pone.0306392.s004.docx]

**
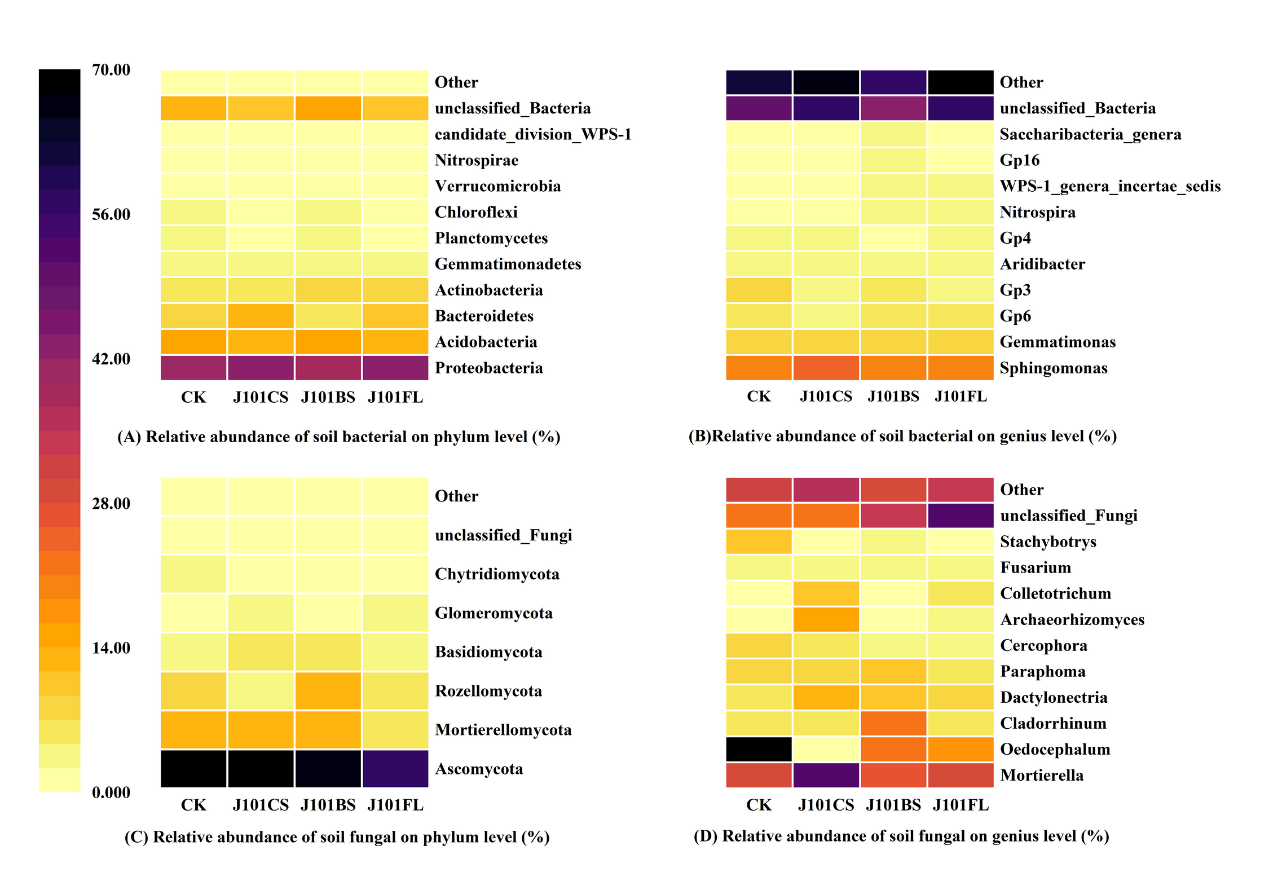
**

**S4 Fig. The relative abundance of bacterial and fungal communities in maize rhizosphere soil.** (A) Changes in the relative abundance of bacteria at the phylum level; (B) changes in the relative abundance of bacteria at the genius level; (C) changes in the relative abundance of fungi at the phylum level; and (D) changes in the relative abundance of fungi at the genius level.
