# Supplementary material for: Pb-resistant Pantoea rwandensis promotes maize’s growth by altering Pb accumulation in biomass and soil Pb immobilization
Source: PLoS One. 2024 Oct 18;19(10):e0306392. doi: 10.1371/journal.pone.0306392 (PMC11488736; doi:10.1371/journal.pone.0306392)
Supplement: S5 Fig — Note: “-s” represents the shoots of maize, “-l” represents maize leaves, and “-r” represents maize roots; F1: water-soluble forms of Pb; F2: ion-exchange forms of Pb; F3: carbonate-bound forms of Pb; F4: humic-acid-bound forms of Pb; F5: iron-manganese-oxide-bound forms of Pb; F6: strong organic forms of Pb; F7: residual forms of Pb; AP: available phosphorus; AN: alkaline nitrogen; ACP: acid phosphatase activity; and URE: urease activity. (DOCX) [file pone.0306392.s005.docx]

**
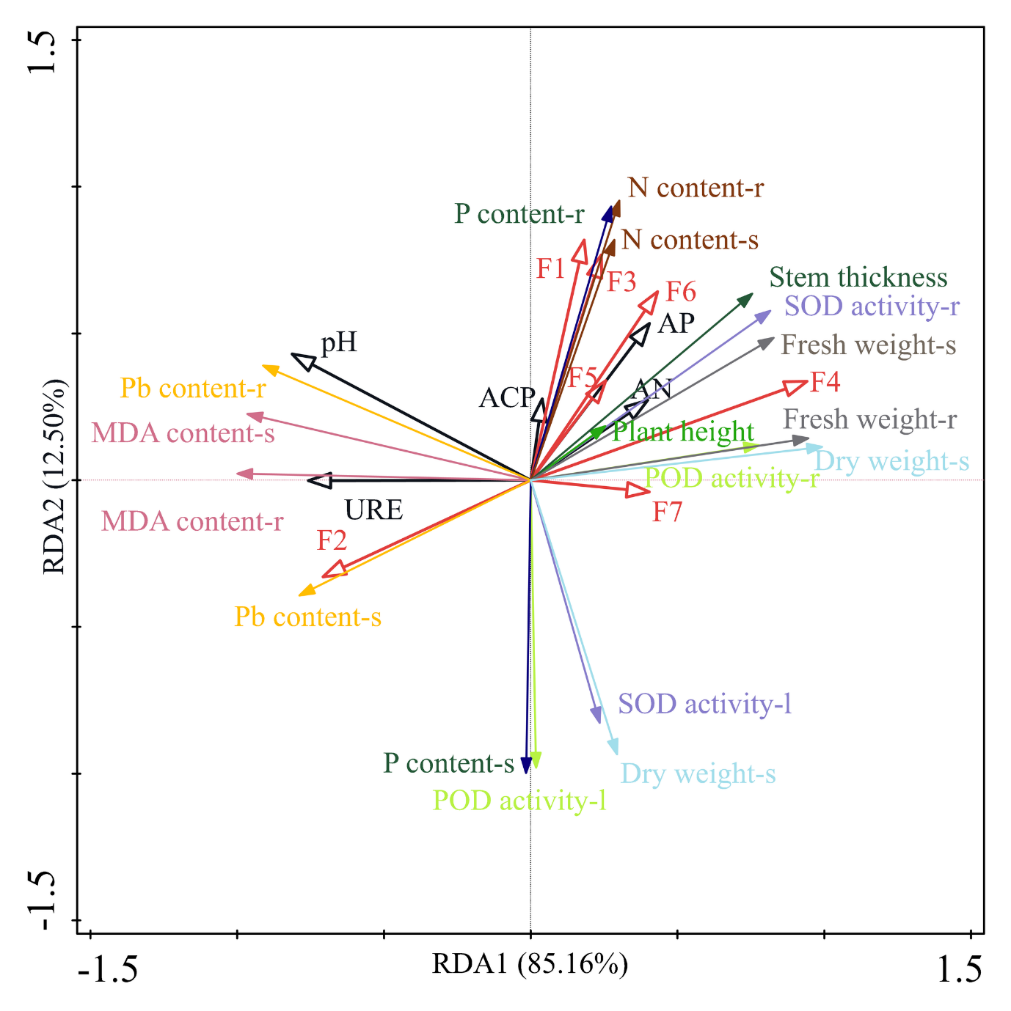
**

**S5 Fig. RDA of maize agronomic traits and biomass in relation to soil physicochemical properties and different forms of Pb. Note:** “-s” represents the shoots of maize, “-l” represents maize leaves, and “-r” represents maize roots; F1: water-soluble forms of Pb; F2: ion-exchange forms of Pb; F3: carbonate-bound forms of Pb; F4: humic-acid-bound forms of Pb; F5: iron-manganese-oxide-bound forms of Pb; F6: strong organic forms of Pb; F7: residual forms of Pb; AP: available phosphorus; AN: alkaline nitrogen; ACP: acid phosphatase activity; and URE: urease activity.
