# Supplementary material for: Pb-resistant Pantoea rwandensis promotes maize’s growth by altering Pb accumulation in biomass and soil Pb immobilization
Source: PLoS One. 2024 Oct 18;19(10):e0306392. doi: 10.1371/journal.pone.0306392 (PMC11488736; doi:10.1371/journal.pone.0306392)
Supplement: S1 Table — Note: Different lowercase letters in the table column-wise indicate significant differences between groups at p< 0.05. (DOCX) [file pone.0306392.s006.docx]

|  | Plant height （cm) | Stem thickness （mm) | Fresh weight of shoot (g) | Fresh weight of roots (g) | Dry weight of shoot (g) | Dry weight of roots (g) |
| --- | --- | --- | --- | --- | --- | --- |
| CK | 26.30±1.64a | 5.29±0.75a | 3.28±0.84a | 2.21±0.59a | 0.57±0.14a | 0.56±0.07a |
| J101CS | 31.25±7.04b | 6.86±1.35b | 5.56±1.36b | 6.08±1.22b | 0.85±0.31b | 0.47±0.05a |
| J101BS | 31.83±7.34b | 6.28±0.72b | 5.78±0.90b | 6.52±0.98b | 0.88±0.08b | 0.66±0.07b |
| J101FL | 31.91±2.55b | 7.00±1.04b | 7.59±1.97c | 9.16±2.79c | 1.19±0.38c | 0.66±0.12b |

**S1 Table.** **Effects of different treatments on agronomic traits and biomass of maize.**

**Note:** Different lowercase letters in the table column-wise indicate significant differences between groups at *p*< 0.05.
