# Supplementary material for: Pb-resistant Pantoea rwandensis promotes maize’s growth by altering Pb accumulation in biomass and soil Pb immobilization
Source: PLoS One. 2024 Oct 18;19(10):e0306392. doi: 10.1371/journal.pone.0306392 (PMC11488736; doi:10.1371/journal.pone.0306392)
Supplement: S2 Table — Note: Different lowercase letters in the table row-wise indicate significant differences between groups at p< 0.05. (DOCX) [file pone.0306392.s007.docx]

| The forms of heavy metal Pb（mg⋅kg^-1^） | CK | J101CS | J101BS | J101FL |
| --- | --- | --- | --- | --- |
| Water-soluble forms | 3.32±0.01a | 3.45±0.00c | 3.39±0.00b | 3.37±0.02b |
| Ion-exchange forms | 168.41±2.05b | 144.69±1.47a | 144.14±0.63a | 145.58±0.89a |
| Carbonate-bound forms | 431.36±1.88a | 700.98±11.91d | 582.58±13.41c | 535.61±24.37b |
| Humic acid-bound forms | 7.44±0.09a | 8.49±0.25b | 8.18±0.18b | 8.04±0.33b |
| Iron-manganese oxide-bound forms | 1064.62±45.63a | 1198.70±15.83b | 1150.18±25.75b | 1276.75±26.08c |
| Strong organic-bound forms | 240.12±2.57a | 335.52±21.88c | 345.80±17.49c | 283.76±22.11b |
| Residual forms | 70.19±3.09a | 104.98±0.38c | 133.23±1.70d | 99.45±2.43b |

**S2 Table. Effects of different treatments on Pb forms in soil.**

**Note:** Different lowercase letters in the table row-wise indicate significant differences between groups at *p*< 0.05.
