# Supplementary material for: Pb-resistant Pantoea rwandensis promotes maize’s growth by altering Pb accumulation in biomass and soil Pb immobilization
Source: PLoS One. 2024 Oct 18;19(10):e0306392. doi: 10.1371/journal.pone.0306392 (PMC11488736; doi:10.1371/journal.pone.0306392)
Supplement: S3 Table — Note: Different lowercase letters in the table column-wise indicate significant differences between groups at p< 0.05. AP: available phosphorus; AN: alkaline nitrogen; ACP: acid phosphatase activity; and URE: urease activity. (DOCX) [file pone.0306392.s008.docx]

|  | URE activity  (mg. g^-1^, 24^-1^) | ACP activity  (mg. g^-1^, 24h^-1^) | AP  (mg.kg^-1^) | AN  (mg.kg^-1^) | pH value |
| --- | --- | --- | --- | --- | --- |
| CK | 18354.70±6379.09a | 270.00±33.00a | 14.40±1.68a | 133.00±9.90a | 7.15± 0.01b |
| J101CS | 15901.97±4259.94a | 365.00±30.64bc | 20.87 ±0.52c | 130.67±38.55a | 7.11± 0.04b |
| J101BS | 14519.37 ±3988.02a | 385.00 ±2.36c | 19.86 ±0.25c | 105.00±0.00a | 6.91± 0.05a |
| J101FL | 15030.87 ±4613.85a | 290.00±42.43±ab | 18.05 ±0.40b | 149.33±29.14a | 6.90± 0.06a |

**S3 Table. Effects of different inoculation on soil properties.**

**Note:** Different lowercase letters in the table column-wise indicate significant differences between groups at *p*< 0.05. AP: available phosphorus; AN: alkaline nitrogen; ACP: acid phosphatase activity; and URE: urease activity.
