# Supplementary material for: Pb-resistant Pantoea rwandensis promotes maize’s growth by altering Pb accumulation in biomass and soil Pb immobilization
Source: PLoS One. 2024 Oct 18;19(10):e0306392. doi: 10.1371/journal.pone.0306392 (PMC11488736; doi:10.1371/journal.pone.0306392)
Supplement: S4 Table — Note: Different lowercase letters in the table column-wise indicate significant differences between groups at p< 0.05. (DOCX) [file pone.0306392.s009.docx]

| Inoculation | Bacterial community | |  | Fungal community | |
| --- | --- | --- | --- | --- | --- |
|  | Chao | Simpson |  | Chao | Simpson |
| CK | 2,765.35± 9.07a | 0.0086±0.0024a |  | 647.85±51.30 a | 0.27±0.17a |
| J101CS | 2,685.36± 65.86a | 0.0112±0.0047a |  | 768.77±17.67b | 0.05± 0.01b |
| J101BS | 2,720.58± 10.36a | 0.0085±0.0004a |  | 743.18±19.82ab | 0.05± 0.01b |
| J101FL | 2,723.07± 39.56a | 0.0077±0.0025a |  | 804.30±44.27b | 0.17± 0.03a |

**S4 Table. The α-diversity index of bacterial and fungal communities in maize rhizosphere soil.**

**Note:** Different lowercase letters in the table column-wise indicate significant differences between groups at *p*< 0.05.
