# Supplementary material for: Pb-resistant Pantoea rwandensis promotes maize’s growth by altering Pb accumulation in biomass and soil Pb immobilization
Source: PLoS One. 2024 Oct 18;19(10):e0306392. doi: 10.1371/journal.pone.0306392 (PMC11488736; doi:10.1371/journal.pone.0306392)
Supplement: Attachment — Submitted filename: PONE-D-23-42620_reviewer.pdf [file pone.0306392.s011.pdf]

PLOS ONE

Pb-resistant phosphate-solubilizing bacteria promote maize's growth by altering Pb accumulation in biomass and soil Pb immobilization

--Manuscript Draft--

Manuscript Number:	PONE-D-23-42620
Article Type:	Research Article
Full Title:	Pb-resistant phosphate-solubilizing bacteria promote maize's growth by altering Pb accumulation in biomass and soil Pb immobilization
Short Title:	phosphate-solubilizing bacteria promote maize's growth by altering soil Pb immobilization
Corresponding Author:	Xiuli Hou, Ph,D.<br>Kunming University<br>CHINA
Keywords:	phosphate-solubilizing bacteria; lead immobilization; growth promotion; microbial community; lead accumulation in biomass
Abstract:	In lead (Pb) contaminated soil, inoculation of phosphate solubilizing bacteria (PSB) to reduce the phytoavailability of Pb and change soil nutrient was an important way to inhibit the Pb uptake by plants. In this study, we isolated the native Pb-resistant Pantoea rwandensis of PSB in tailing site and employed broth culture and pot experiments to investigate the effect of inoculation P. rwandensis on soil Pb immobilization, soil nutrient, the microbial community and Pb accumulation in biomass of maize (Zea mays L.). The results showed that Pb-resistant P. rwandensis not only had a certain tolerance to Pb2+, but also could solubilize Ca3(PO4)2 by secreting oxalic acid, tartaric acid, lactic acid, and acetic acid. It significantly increases the amount of soluble P, which reached 567.50 mg.L-1. In the pot experiment, the application of clear supernatant, inoculation with P. rwandensis, the mixture of supernatant and P. rwandensis increased soil residual Pb by 49.57%, 89.81%, and 41.69%, respectively, comparing to the control. Consequently, the increase of residual Pb in soil inhibited its uptake by maize, especially inoculated P. rwandensis and a mixture of supernatant and P. rwandensis decreased the accumulation Pb in aboveground biomass of maize by 61.65% and 72.48%, and in roots of maize by 26.00% and 39.59%. Meanwhile, P. rwandensis and the mixture of supernatant and P. rwandensis increased aboveground biomass of maize by 54.39%, 17.86%, and root of maize by 108.77%, and 17.86%, respectively. P. rwandensis inoculation promote the biodiversity of the fungal community, and increases the abundance of Proteobacteria, Mortierella, and Sphingomonas, which have high capacity of fixation and absorption to heavy metal. Our results demonstrated that inoculation with P. rwandensis could promote the transformation of Pb fractions, together with soil bacteria community function change, ultimately decreasing Pb accumulation in biomass to promote the development of maize.
Order of Authors:	Runhai Jiang
	Chengqiang Zhu
	Shaofu Wen
	Mei Zhang
	Yani Zhang
	Xiuli Hou
Additional Information:	
Question	Response
Financial Disclosure<br><br>Enter a financial disclosure statement that	The author(s) received no specific funding for this work.

Powered by Editorial Manager® and ProduXion Manager® from Aries Systems Corporation

describes the sources of funding for the work included in this submission. Review the submission guidelines for detailed requirements. View published research articles from *PLOS ONE* for specific examples.<br><br>This statement is required for submission and **will appear in the published article** if the submission is accepted. Please make sure it is accurate.<br><br>**Funded studies**<br>Enter a statement with the following details:<br>• Initials of the authors who received each award<br>• Grant numbers awarded to each author<br>• The full name of each funder<br>• URL of each funder website<br>• Did the sponsors or funders play any role in the study design, data collection and analysis, decision to publish, or preparation of the manuscript?<br><br>Did you receive funding for this work?	
**Competing Interests**<br><br>Use the instructions below to enter a competing interest statement for this submission. On behalf of all authors, disclose any competing interests that could be perceived to bias this work—acknowledging all financial support and any other relevant financial or non-financial competing interests.<br><br>This statement is required for submission and **will appear in the published article** if the submission is accepted. Please make sure it is accurate and that any funding sources listed in your Funding Information later in the submission form are also declared in your Financial Disclosure statement.<br><br>View published research articles from *PLOS ONE* for specific examples.	The authors have declared that no competing interests exist.

**NO authors have competing interests**<br><br>Enter: *The authors have declared that no competing interests exist.*<br><br>**Authors with competing interests**<br><br>Enter competing interest details beginning with this statement:<br><br>*I have read the journal's policy and the authors of this manuscript have the following competing interests: [insert competing interests here]*<br><br>* typeset	
**Ethics Statement**<br><br>Enter an ethics statement for this submission. This statement is required if the study involved:<br><br>• Human participants<br>• Human specimens or tissue<br>• Vertebrate animals or cephalopods<br>• Vertebrate embryos or tissues<br>• Field research<br><br>Write "N/A" if the submission does not require an ethics statement.<br><br>General guidance is provided below. Consult the submission guidelines for detailed instructions. **Make sure that all information entered here is included in the Methods section of the manuscript.**	None

Powered by Editorial Manager® and ProduXion Manager® from Aries Systems Corporation

**Format for specific study types**<br><br>**Human Subject Research (involving human participants and/or tissue)**<br>• Give the name of the institutional review board or ethics committee that approved the study<br>• Include the approval number and/or a statement indicating approval of this research<br>• Indicate the form of consent obtained (written/oral) or the reason that consent was not obtained (e.g. the data were analyzed anonymously)<br><br>**Animal Research (involving vertebrate animals, embryos or tissues)**<br>• Provide the name of the Institutional Animal Care and Use Committee (IACUC) or other relevant ethics board that reviewed the study protocol, and indicate whether they approved this research or granted a formal waiver of ethical approval<br>• Include an approval number if one was obtained<br>• If the study involved *non-human primates*, add *additional details* about animal welfare and steps taken to ameliorate suffering<br>• If anesthesia, euthanasia, or any kind of animal sacrifice is part of the study, include briefly which substances and/or methods were applied<br><br>**Field Research**<br><br>Include the following details if this study involves the collection of plant, animal, or other materials from a natural setting:<br>• Field permit number<br>• Name of the institution or relevant body that granted permission	
**Data Availability**<br><br>Authors are required to make all data underlying the findings described fully available, without restriction, and from the time of publication. PLOS allows rare exceptions to address legal and ethical concerns. See the PLOS Data Policy and FAQ for detailed information.	Yes - all data are fully available without restriction

A Data Availability Statement describing where the data can be found is required at submission. Your answers to this question constitute the Data Availability Statement and **will be published in the article**, if accepted.<br><br>**Important:** Stating 'data available on request from the author' is not sufficient. If your data are only available upon request, select 'No' for the first question and explain your exceptional situation in the text box.<br><br>Do the authors confirm that all data underlying the findings described in their manuscript are fully available without restriction?	
**Describe where the data may be found in full sentences. If you are copying our sample text, replace any instances of XXX with the appropriate details.**<br><br>• If the data are **held or will be held in a public repository**, include URLs, accession numbers or DOIs. If this information will only be available after acceptance, indicate this by ticking the box below. For example: *All XXX files are available from the XXX database (accession number(s) XXX, XXX.).*<br>• If the data are all contained **within the manuscript and/or Supporting Information files**, enter the following: *All relevant data are within the manuscript and its Supporting Information files.*<br>• If neither of these applies but you are able to provide **details of access elsewhere**, with or without limitations, please do so. For example:<br><br>*Data cannot be shared publicly because of [XXX]. Data are available from the XXX Institutional Data Access / Ethics Committee (contact via XXX) for researchers who meet the criteria for access to confidential data.*<br><br>*The data underlying the results presented in the study are available from (include the name of the third party*	All relevant data are within the manuscript and its Supporting Information files.

and contact information or URL).<br>• This text is appropriate if the data are owned by a third party and authors do not have permission to share the data.<br><br>* typeset	
Additional data availability information:	

Powered by Editorial Manager® and ProduXion Manager® from Aries Systems Corporation

Manuscript Click here to access/download;Manuscript;Manuscript.docx

Pb-resistant phosphate-solubilizing bacteria promote maize's growth by altering Pb accumulation in biomass and soil Pb immobilization

Runhai Jiang[1], Chengqiang Zhu[1], Shaofu Wen[1], Mei Zhang[1], Yani Zhang[1], Xuexiu Chang[1], Xiuli Hou[1*]

[1] Yunnan Collaborative Innovation Center for Plateau Lake Ecology and Environmental Health (funded by Yunnan Provincial Education Department), College of Agronomy and Life Sciences, Kunming University, Kunming, Yunnan, China

*Corresponding author:

Email address: hxlyn@aliyun.com (XLH).

Abstract

In lead (Pb) contaminated soil, inoculation of phosphate solubilizing bacteria (PSB) to reduce the phytoavailability of Pb and change soil nutrient ~~was~~ an important way to inhibit the Pb uptake by plants. In this study, we isolated the native Pb-resistant *Pantoea rwandensis* ~~of PSB in~~ tailing site and employed broth culture and pot experiments to investigate the effect of inoculation *P. rwandensis* on soil Pb immobilization, soil nutrient, the microbial community and Pb accumulation in biomass of maize (*Zea mays* L.). The results showed that ~~Pb-resistant~~ *P. rwandensis* not only ~~had a certain tolerance to~~ Pb^{2+}, but also ~~could solubilize~~ $Ca_3(PO_4)_2$ by secreting oxalic acid, tartaric acid, lactic acid, and acetic acid. It significantly increases the amount of soluble P, which reached 567.50 mg·L^{-1}. In the pot experiment, the application of clear supernatant, inoculation with *P. rwandensis*, the mixture of supernatant and *P. rwandensis* increased soil residual Pb by 49.57%, 89.81%, and 41.69%, respectively, ~~comparing~~ to the control. Consequently, the increase of residual Pb in soil inhibited its uptake by maize, especially inoculated *P. rwandensis* and a mixture of supernatant and *P. rwandensis* decreased the accumulation Pb in aboveground biomass of maize by 61.65% and 72.48%, and in roots ~~of maize~~ by 26.00% and 39.59%. Meanwhile, *P. rwandensis* and the mixture of supernatant and *P. rwandensis* increased aboveground biomass of maize by 54.39%, 17.86%, and root of maize by 108.77%, and 17.86%, respectively. *P. rwandensis* inoculation ~~promote~~ the biodiversity of the fungal community, and ~~increases~~ the abundance of *Proteobacteria*, *Mortierella*, and *Sphingomonas*, which have high capacity of fixation and absorption to heavy metal. Our results demonstrated that inoculation with *P. rwandensis* could promote the transformation of Pb fractions, together with soil bacteria community function change, ultimately decreasing Pb accumulation in biomass to promote the development of maize.

Keywords: phosphate-solubilizing bacteria; lead immobilization; growth promotion; microbial community; lead accumulation in biomass

Introduction

~~Heavy metal soil~~ contamination with lead (Pb) is a global environmental issue because of mining and smelting [1, 2]. Its toxicity and enrichment in plants and animals have been attracted more attention, so chemical precipitation, absorption, and ion exchange to immobilize Pb in soil are the effective methods for decreasing uptake and accumulation in plants [3-5]. According to the ~~BCR (~~European Community Bureau of Reference~~)~~ sequential extraction method, heavy metal has been divided into four speciation: soluble/exchangeable fraction (F1), reducible fraction (F2), oxidizable fraction (F3), and residual fraction (F4), and the F4 was recognized as the most stable fraction. Nowadays, several phosphate-based amendments such as dicalcium phosphate, diammonium phosphate, mono-ammonium phosphate, phosphoric acid, apatite, and hydroxyapatite ~~have already been~~ applied to promote the transformation of Pb from unstable fraction (F1, F2 and F3) to mineral structure (F4) for immobilization remediation of Pb-contaminated soil [6, 7]. The phosphate-based amendments would induce ~~to form~~ various Pb-phosphorate compounds such as $Pb_5(PO_4)_3X$ [X=F (fluorine), Cl (chlorine), Br (bromine), OH (hydroxy)] in ~~Pb-contaminated soil, which its bioavailability would be reduced.~~ But some factors such as the solubility of phosphorus (P) sources, the pH value, the Pb speciation, and particle size could ~~affect each other~~ and make Pb-immobilization formation more difficult. To ensure the high solubility of P, the excessive dose of P-compounds ~~was~~ used in soil to ensure sufficient soluble orthophosphate anions for Pb immobilization [8]. But the excessive amount of P-compound may ~~make~~ eutrophication ~~in~~ water environments [9-12]. So, it is a hot issue to environmentally friendly improve the P solubility for Pb immobilization in Pb contaminated soil.

PSB are widely distributed in soil, which could drive P transformation and dominate the composition of P fractions. Inorganic phosphorus compounds are solubilized by PSB mainly through the production of organic acids, which reduces the pH of rhizosphere soil or chelates the cations that are responsible for P precipitation [13-

16]. In addition, PSB can enhance soil acid phosphatase and alkaline phosphatase through enzymatic hydrolysis to mineralize soil organic P [17, 18]. So many studies inculcated PSB can promote plant growth by increasing the bioavailable P level in soil and produce plant-beneficial metabolites such as phytohormones and siderophores, especially in infertile soil [19-21]. Apart from their functional roles in increasing the amount of soil available P, the increase of soluble P in soil provides the conditions for the formation of Pb-phosphate compounds in Pb-contaminated soil due to the accelerated release of phosphate from native insoluble P species in soil (mainly Fe–P). Currently, PSB was used to immobilize Pb by its mineralizing phosphate [22]. Pb immobilization in soil inhibited Pb uptake by plants and decreased the toxicity of Pb [23-25].

In the past decades, farmers applied amount of fertilizer into Pb-contaminated agricultural fields and insisted that enough nutrient supply could promote the growth of crops, especially in the Jinding Pb-Zn mining area, Yunnan Province, southwest of China, which is the highest reserves of Zn in Asia [26, 27]. Large amounts of P applied as fertilizer were transformed into various unavailable forms through precipitation with the highly reactive Al^{3+} and Fe^{3+} in soil [28]. Therefore, inoculated Pb-resistant PSB in Pb-contaminated soil to facilitate P uptake by plants and immobilization of Pb is the effective way for sustainable development of local people. At present, the single aspect has been focused on the effects of PSB on plant growth, soil P conversion mechanisms, Pb-immobilization, and microbial community structures of soil [29-31]. Given the function of PSB on mobilization Pb, the transformation of soil P, and soil nutrient changes in most Pb-contaminated soils, we hypothesize that the inoculation of Pb-resistant PSB to Pb-contaminated soils could promote plant growth by immobilization of soil Pband decreasing accumulation Pb in biomass. The objectives of this study were to (i) investigate the ability of the native PSB in solubilizing $Ca_3(PO_4)_2$ and tolerance to Pb^{2+} in broth culture conditions, (ii) examine the effects of Pb-resistant PSB on Pb immobilization and soil microbial community structure under soil conditions, and (iii)

determine whether Pb-resistant PSB could promote plant growth by reducing the accumulation of Pb in biomass of maize and changing oxidative stress indicators. Results from this study should understand the relationships among plant growth, Pb immobilization, and soil microbial communities.

Materials and methods

Screening of PSB

~~The samples of soil in this~~ study were collected from Jinding Pb-Zn tailing site, located Nujiang Lisu Autonomous Prefecture, Yunnan Province, Southwest of China. The geographical location is 26°27'37.36"N and 99°28'32.25"E, with an altitude of 2785 m. ~~10 Plants~~ growing well were selected in the tailings and the soil samples were collected from the rhizosphere soil of these plants. Then, the samples were placed into sterile bags and transported to the laboratory as soon as possible. The samples are stored at 4°C and used for strain screening.

Suspended 2 g of soil were taken from the stored soil samples and added to a 100 mL triangular flask with 18 mL of sterile water. The mixture was shaken on an orbital shaker at 180 rpm·min^{-1} for 30 min (28°C). From this, 1 mL suspension was taken and added to 9 mL of sterile water, which was further diluted to create suspensions with soil dilutions of 10^{-4}, 10^{-5}, and 10^{-6}. The PSB were screened and isolated by Pikovskaya (PVK) medium consisting of the following constituents: glucose 10 g, $Ca_3(PO_4)_2$ 5 g, $(NH_4)_2SO_4$ 0.5 g, NaCl 0.3 g, $MgSO_4{\cdot}7H_2O$ 0.3 g, KCl 0.3 g, $MnSO_4$ 0.02 g, $FeSO_4{\cdot}7H_2O$ 0.03 g, agar 18 g, distilled water 1000 mL, pH 7.0, Sterilize at 115°C for 30 minutes [32]. Then, 0.25 mL of each dilution were spread onto PVK solid medium plates and incubated at 28°C for 4-7 days after clear halo zones were formed around the bacteria and the single colonies were distinct without linking to each other. The PSB was isolated with clear zones, then it was transferred to Luria-Bertan (LB) medium. The PSB were purified using the streak plate method to acquire single colonies, then it was maintained on LB medium plates at 4°C.

Acid production and phosphate solubilization of bacteria

The screening strain PSB was activated and cultured in LB liquid medium at 180 rpm·min^{-1} for 30 min (28°C). The bacterial suspension was adjusted to approximately 1×10^8 CFU·mL^{-1} concentration of strain using sterile water. Then, 1 mL of the bacterial suspension was inoculated into a PVK broth medium containing 1 g $Ca_3(PO_4)_2$ per 100 mL, which served as the sole P source. In the control treatment, all components were the same except that sterile water was used instead of the PSB suspension. The fermentation liquid was continuously incubated at 180 rpm·min^{-1} for 7 days (28°C). A sample of the fermentation liquid (20 mL) was taken every day from each flask, centrifuged, and filtered through a 0.45 mm filter membrane. The pH value of the filtrate was measured, and the soluble phosphate in the filtrate was determined using the molybdenum antimony anti-colorimetric method [33]. The dissolution rate of Ca_3 $(PO_4)_2$ was calculated by the following formula.

$$c\ (\%) = \frac{m}{M} \times 100\%$$

c: the dissolution rate of $Ca_3(PO_4)_2$ (%);

m: the content of soluble phosphorus in fermentation liquid (mg/L);

M: the content of $Ca_3(PO_4)_2$ in fermentation liquid (mg/L).

At the 7th day, the fermentation liquid was taken to measure organic acid by high performance liquid chromatography [34]. The PSB suspension was diluted with sterile water to achieve a concentration of 1×10^8 CFU·mL^{-1}. 1 mL of the PSB suspension was incubated to 100 mL of LB-modified liquid medium with 5 mM·L^{-1} tryptophan (precursor for IAA biosynthesis) and cultured at 180 rpm·min^{-1} for 3 days (28°C). This fermentation liquid was tested for IAA by UV visible spectrophotometer (DR 5000, USA) [35].

PSB's tolerance to Pb

Firstly, 1 mL of strain suspension, which contains about 1×10^8 CFU·mL^{-1}, was added to a PVK broth medium.

In the control treatment, all components in medium were the same except that sterile water was used instead of the PSB suspension. $Pb(NO_3)_2$ was applied in fermentation liquid, and the different concentration of Pb^{2+} was 0, 250, 500, 750, and 1000 $mg·L^{-1}$. This PSB was incubated in t[illegible]ermentation liquid with the different concentration of Pb^{2+}. After 7 days culture at 180 $rpm·min^{-1}$ (28°C). With the control treatment as the reference, the OD600 value of the bacterial liquid was determined at 600 nm using a UV visible spectrophotometer (DR 5000, USA).

Microbiological identification of bacteria

This Pb resistant PSB above was labeled J101. The extract genomic DNA of J101 extracting by bacterial total DNA extraction reagent. Primers 27F (5’-AGTTTGATCMTGGCTCAG-3’) and 1492R (5’-GGTTACCTTGTTACGACTT-3’) were used for PCR amplification of 16S rRNA gene [36]. The amplified products were quantified using 1% agarose gel electrophoresis and sequenced by Sangon Biotech Co., Ltd (Shanghai, CHN). The sequences were aligned to the GenBank database using the NCBI Basic local alignment search tool (BLAST) (https://blast.ncbi.nlm.nih.gov/Blast.cgi). A phylogenetic tree was constructed using the MEGA software (version 11; Mega, Raynham, MA, USA).

Pot experiment

The soil for pot experiment was collected from the top layer (0-20 cm) of farmland located near Jinding Pb-Zn tailing site. The background indicators of soil is total phosphorus (TP): 860.33±2.52 $mg·kg^{-1}$, available phosphorus (AP): 12.07±3.70 $mg·kg^{-1}$, alkaline hydrolysis nitrogen (AN): 65.33±4.04 $mg·kg^{-1}$, Pb content: 1985.47 $mg·kg^{-1}$, pH:7.61±0.00. In pot experiment, the seeds of maize "Stadatian 221" were firstly steeped in 95% ethanol for 30 s before being washed 3 times with distilled water. Secondly, these seeds were planted in seedling trays to germinate. After 7 days, we chose two healthy plants with similar development and placed them into

18×15×14 cm pot container. Plastic film was placed at the bottom of each pot to prevent metal and nutrient loss. Each container was filled with 2.5 kg of soil.

After J101 was cultured in LB liquid medium at 180 rpm·min^{-1} for 72 h (28°C), sterile water was added to the fermentation liquid for reach a viable cell density of 1×108 CFU·mL^{-1}. The remaining fermentation liquid was centrifuged at 4°C and 5000 rpm for 10 min and the clear supernatant was collected. The bacterial cells were washed 3 times with sterile physiological saline (0.9% sodium chloride) and then adjusted to a viable cell density of 1×10^{8} CFU·mL^{-1} using sterile water. The clear supernatant contained only secretions from J101, while the bacteria solution contained the bacteria J101. The fermentation liquid was a mixture of both the clear supernatant and J101. We applied the clear supernatant, bacteria solution, and the combination of clear supernatant and bacteria solution separately in maize rhizosphere soil and investigate whether J101 plays a significant role in immobilizing Pb or promoting maize growth through their metabolites or bacterial cells.

In pot experiment, each treatment was replicated 3 times. In maize rhizosphere soil, the clear supernatant treatment was applied (J101CS), the bacteria solution was inoculated (J101BS), and the fermentation liquid was inoculated (J101FL). Experimental pots were watered 300 mL of clear supernatant, bacteria solution, and fermentation liquid from J101 around the maize rhizosphere soil. The control treatment pots were replaced with an equal amount of sterile water. The experiment was conducted in a greenhouse with daytime/nighttime temperatures of 30°C /25°C. Watering occurred every 3 days, and the experiment lasted for 45 days.

Plant and soil chemical analysis

We collected soil samples from 1-2 cm near the maize roots for analysis of soil microbial community structure. Sequencing was accomplished by the Sangon Company (Shanghai, China) based on the manufacturer's instructions by using the llumina MiSeg system (llumina MiSeq, USA).   Another soil samples were air-dried,

ground, and sieved through a 0.149 mm sieve to analyze of soil characteristics. The soil alkaline nitrogen (AN) and soil available phosphorus (AP), urease activity (URE), acid phosphatase activity (ACP), and pH were measured according to soil agrochemical analysis method [37]. The different Pb fractions in soil was determined by extracting solutions according to China Geological Survey's seven-step continuous extraction method (D2005-03, (cgs.gov.cn)).

The maize plants were harvested after 45 days, their aboveground parts and roots are separated and rinsed thoroughly with deionized water. The length and fresh biomass of both parts are recorded, and the root is scanned using a root scanner (Microtek Scanmaker i800 plus, CHN). The Malondialdehyde (MDA) content, superoxide dismutase (SOD) and peroxidase (POD) of maize leaves and roots were measured using reagent kits provided by the Nanjing Institute of Biotechnology Engineering. For dry weight measurement, the samples of maize were dried in an oven at 105°C for 30 min and then reached constant weight by removing moisture at 85°C. then and the dried samples of plants are crushed into a fine powder with a particle size of less than 0.149 mm to determine nitrogen (N), P, and Pb contents according to the analysis method [37].

Data analysis

Statistical analyses in the study were performed using SPSS 27.0. One-way ANOVA analysis of variance was conducted to compare the means derived from different treatment. Significance was calculated by Duncan’s multiple range test at the $p < 0.05$ level. The 16S rRNA sequences were compared by BLAST program, and then the phylogenetic tree was constructed by MEGA 11.0 software. Canoco 5.0 was used to analyze the correlation among antioxidant enzyme activity, N, P, and Pb accumulation in maize leaves and roots, and maize biomass indicators. Soil microbial classification data were analyzed on an online cloud platform (ngs.sangon.com).

Results

P solubilization and Pb tolerance of PSB

Inoculation with strain J101 increased the amount of dissolved P in the fermentation with incubation time (Fig. 1A). The highest amount of dissolved P was observed in the fermentation liquid after 4 days of inoculation with strain J101, with the value of 567.50 mg·L^{-1}, resulting in a dissolution rate of $Ca_3(PO_4)_2$ at 56.75%. However, after 5 days, the amount of dissolved P showed a decreasing trend. Meanwhile, there was a significant decrease in pH in the fermentation liquid over time, with the lowest pH value of 3.94 after 4 days. Pearson correlation analysis revealed a highly significant negative correlation between the amount of dissolved P and pH (r = -0.925**, p<0.01).

When the PSB J101 was grown in fermentation liquid with different concentrations (0, 250, 500, 750, and 1000 mg·L^{-1}) of Pb^{2+}, we found that PSB J101 were inhibited with the high Pb^{2+} concentration (Fig. 1B). However, at the concentration of 250 mg·L^{-1} Pb^{2+}, the PSB J101 showed 40.6% of its activity without Pb in the fermentation liquid. When this strain was exposed to the concentration of 750 mg·L^{-1} Pb^{2+}, it could maintain growth although its OD_{600} values decreased, indicating that J101 has a certain tolerance to Pb^{2+}.

The organic acids secreted by PSB were responsible for dissolved P from $Ca_3(PO_4)_2$ (Fig. 1C). When $Ca_3(PO_4)_2$ was the sole source of P in fermentation liquid s, the PSB J101 secreted oxalic acid, tartaric acid, lactic acid and acetic acid with the values of 429.51 mg·L^{-1}, 71.85 mg·L^{-1}, 271.4 mg·L^{-1}, and 374.43 mg·L^{-1} respectively. These results indicated that this strain secretes organic acids to lower the pH in the culture medium, and $Ca_3(PO_4)_2$ in the medium is dissolved with the lowering of the environmental pH. Moreover, J101 secretes IAA with a value of 34.14 mg·L^{-1}(Fig. 1C), which promotes plant growth. This result indicated that the PSB J101 has a potential growth-promoting effect on plants by secreting a certain amount of IAA.

Fig 1. This is the Fig 1 Title. The change of soluble phosphorus content and pH value in fermentation liquid (A). The OD values at 600nm after culturing the PSB in fermentation liquid with Pb^{2+} concentrations of 0, 250, 500, 750, and 1000 mg·L^{-1} for 72 h (B). The content of organic acid and IAA secreted by the PSB (C).

l6S rDNA gene of PSB

A 1478-bp 16S rRNA gene fragment was obtained from strain J101. A phylogenetic tree was constructed based on the alignment results compared with data in GenBank, showing that the sequence had a high similarity of 98.81% with *Pantoea rwandensis* strain (NR118121.1). Therefore, the strain was identified as *P. rwandensis* (Fig. S1).

Maize's growth-promoting by PSB

After application of clear supernatant (J101CS), inoculation with bacteria (J101BS), and inoculation with the mixture of supernatant and bacteria (J101FL) in Pb-contaminated soil, the height, stem thickness, fresh weight of aboveground and roots, and dry weight of aboveground and roots in maize significantly increased compared to the un-inoculation treatment group (Table 1). Inoculation of bacteria (J101BS) and the mixture of supernatant and bacteria (J101FL) increased these six parameters of maize by 21.03%, 18.71%,76.22%, 195.02%, 54.39%, 17.86%, and 21.33%, 32.33%, 131.40%, 314.48%, 108.77%, 17.86%, respectively. Except for the dry weight of roots, the plant height, stem thickness, fresh weight of aboveground, fresh weight of roots, dry weight of aboveground, and dry weight of roots of maize in the J101CS treatment group increased by 18.82%, 29.68%, 69.51%, 175.11%, and 49.12%, respectively. Moreover, the root biomass of maize showed an improvement in J101CS, J101BS, and J101FL treatment groups (Fig. S2).

Table 1. This is the Table 1 Title. Changes of biomass in maize. CK: non-inoculated, J101CS: application with clear supernatant, J101BS: inoculated with the bacteria, J101FL: inoculated with the mixture of supernatant and

bacteria. One-way analysis of variance was performed followed by Duncan's multiple range test at a significance level of *p*<0.05. Different letters indicate statistically significant differences.

The enrichment of lead and change of antioxidant enzyme activity in maize

Application of the clear supernatant (J101CS), inoculation with bacteria (J101BS) and the mixture of supernatant and bacteria (J101FL) reduced the accumulation of Pb in maize leaves by 69.40%, 61.65%, and 72.48%, respectively, compared to the un-inoculation treatment group (Fig. 2A). Inoculation with J101BS and J101FL also significantly decreased the accumulation of Pb in maize roots by 26.00% and 39.59%, respectively. The SOD activity in maize leaves decreased by 11.79% in J101CS treatment, compared to un-inoculation treatment group. Inoculation with bacteria (J101BS), and the mixture of supernatant and bacteria (J101FL) increased the SOD activity in maize roots by 9.46%, 3.71%, and 9.86% respectively (Fig. 2B), and increased the POD activity in maize roots by 4.58%, 6.25%, and 2.27% respectively, compared to un-inoculation treatment group (Fig. 2C). In J101BS and J101FL treatment groups, the MDA content in maize leaves was significantly lower than in the control group. The MDA content of maize roots showed a significant decrease in J101CS, J101BS and J101FL treatments, especially the lowest value of MDA content was observed in J101FL treatment group with the value of 3.66 nmol·g·prot^{-1}, 95.66% lower than that of un-inoculation treatment group (Fig. 2D).

Fig 2. This is the Fig 2 Title. The content of Pb (A), SOD (B), POD (C) activity and MDA (D) content in maize plants. This is the Fig 1 Title. SOD: superoxide dismutase, POD: peroxidase, MDA: malondialdehyde, CK: non-inoculated, J101CS: application with clear supernatant, J101BS: inoculated with the bacteria, J101FL: inoculated with the mixture of supernatant and bacteria. One-way analysis of variance was performed followed by Duncan's multiple range test

at a significance level of $p<0.05$. Different letters indicate statistically significant differences.

Nutrients uptake by maize

In J101CS and J101FL treatment groups, the N content in maize aboveground and roots significantly increased compared to the un-inoculation treatment group (Fig. 3A), and the N content in aboveground and roots increased by 88.89% and 37.04%, 80.95% and 35.71%, respectively. The P content in maize aboveground was not significantly different among the three treatment groups. However, in J101CS treatment group, the P content in maize roots increased by 23.58% compared to the CK group, although the difference was not significant (Fig. 3B).

Fig 3. This is the Fig 3 Title. The P content, N content, and Pb content in aboveground and roots biomass of maize. CK: non-inoculated, J101CS: application with clear supernatant, J101BS: inoculated with the bacteria, J101FL: inoculated with the mixture of supernatant and bacteria. One-way analysis of variance was performed followed by Duncan's multiple range test at a significance aboveground of $p<0.05$. Different letters indicate statistically significant differences.

Soil properties of maize rhizosphere

Heavy metal has been divided into 7 fractions in soil: water-soluble, ion-exchange, carbonate-bound, humic acid-bound, iron-manganese oxide-bound, strong organic-bound, and residual forms, and the mobility of these 7 fractions of Pb is decreased in the order of water-soluble>ion-exchange>carbonate-bound>humic acid-bound>iron-manganese oxide-bound>strong organic-bound>residual forms. We found that application of clear supernatant (J101CS), inoculation with bacteria (J101BS) and the mixture of supernatant and bacteria (J101FL) could change the soil Pb fractions. In these three treatment groups, the content of ion-exchange Pb in maize

rhizosphere soil was reduced by 14.08%, 14.41%, and 13.56%, respectively (Table 2). However, the fraction of carbonate-bound, humic acid-bound, iron-manganese oxide-bound, and strong organic-bound Pb in maize rhizosphere soil was significantly increased. In the J101CS, J101BS, and J101FL treatment groups, the carbonate-bound Pb content was increased by 62.50%, 35.06%, and 24.17%; the content of humic acid-bound Pb was increased by 14.11%, 9.95%, and 8.06%, the iron-manganese oxide-bound lead was increased by 12.59%, 8.04%, and 19.93%, and the strong organic-bound Pb increased by 39.73%, 44.01%, and 18.17% respectively. Especially, the fraction of residual lead content in maize rhizosphere soil increased significantly by 49.57%, 89.81%, and 41.69% in J101CS, J101BS, and J101FL treatment groups. These results indicated that PSB helped transform the mobilization fraction Pb into immobilization fraction Pb.

Table 2. This is the Table 2 Title. The soil Pb forms in maize rhizosphere. CK: non-inoculated; J101CS: application with clear supernatant, J101BS: inoculated with the bacteria, J101FL: inoculated with the mixture of supernatant and bacteria. One-way analysis of variance was performed followed by Duncan's multiple range test at a significance level of $p<0.05$. Different letters indicate statistically significant differences.

In the pot experiment, we found that the application of clear supernatant (J101CS) and inoculation with bacteria (J101BS) significantly increased the activity of acid phosphatase activity (ACP) in maize rhizosphere soil by 35.19% and 42.59% respectively, compared to the un-inoculation treatment group. Application of clear supernatant (J101CS), inoculation with bacteria (J101BS), and the mixture of supernatant and bacteria (J101FL) also significantly increased the content of AP in the soil by 44.93%, 37.92%, and 25.35% respectively (Table 3). The application of clear supernatant did not significantly affect the pH of maize rhizosphere soil. However inoculation bacteria (J101BS) and the mixture of supernatant and bacteria (J101FL) significantly decreased the

pH values of maize rhizosphere soil compared to the un-inoculation treatment group. Application of clear supernatant (J101CS), and inoculation with bacteria (J101BS) and the mixture of supernatant and bacteria (J101FL) did not significantly affect URE activity and AN in maize rhizosphere soil.

Table 3. This is the Table 3 Title. Soil properties of maize rhizosphere. CK: un-inoculation treatment. J101CS: application with clear supernatant, J101BS: inoculated with the bacteria, J101FL: inoculated with the mixture of supernatant and bacteria One-way analysis of variance was performed followed by Duncan's multiple range test at a significance level of $p<0.05$. Different letters indicate statistically significant differences.

Rhizosphere soil microbial community diversity and structure

PSB can change the diversity and composition of soil microbial communities. In Pb-contaminated soils, there were no significant differences in Chao and Simpson indices of bacterial communities in maize rhizosphere soil in J101CS, J101BS and J101FL treatment groups, compared to un-inoculation treatment (Table 4). However, the Chao index of fungal communities significantly increased in J101CS and J101FL treatment groups, while the Simpson index significantly decreased after application of clear supernatant (J101CS) and inoculation with bacteria (J101BS) (Table 4), indicating that the application of microbial inoculants can significantly enhance the diversity of fungal communities in maize rhizosphere soil.

Table 4. This is the Table 4 Title. The α-diversity indices of bacterial and fungal communities in maize rhizosphere soil. CK: non-inoculated, J101CS: application with clear supernatant, J101BS: inoculated with the bacteria, J101FL: inoculated with the mixture of supernatant and bacteria. One-way analysis of variance was performed followed by Duncan's multiple range test at a significance level of $p<0.05$. Different letters indicate statistically significant differences.

Bacteria from 10 phyla and fungi from 6 phyla were annotated, and the abundance of the top ten types at the phylum level and genus level were analyzed (Fig. 4). In maize rhizosphere soil, *Proteobacteria* was the dominant phylum. The relative abundance of this *Proteobacteria* phylum in maize rhizosphere soil in J101CS and J101FL treatment groups was significantly higher than that of the un-inoculation treatment group, followed by the relative abundance of *Acidobacteria* (Fig. 4A). At the genus level, J101CS and J101FL increased the dominant genus *sphingomonas* and the subdominant *gemmatimonas* abundance of the bacterial community, compared to the un-inoculation treatment group (Fig. 4B). In fungal communities of maize rhizosphere soil, *Ascomycota* was identified as the dominant phylum with a relative abundance range of 59.52% to 71.48%. The relative abundance of *Ascomycota* in J101BS and J101FL treatment groups was lower than that of the un-inoculation treatment group. However, there was a significant decrease in *Mortierellomycota*'s relative abundance in J101FL treatment group (Fig. 4C). At the genus level, *Oedocephalum* was identified as the dominant genus in maize rhizosphere soil with a relative abundance range of 8.27% to 31.91%, followed by *Mortierella* with a relative abundance range of 12.05% to 23.73% (Fig. 4D). Compared to the un-inoculation treatment group, the application of clear supernatant (J101CS) increased the relative abundance of *Mortierella* in maize rhizosphere soil. But in J101CS, J101BS, and J101FL treatment groups, the relative abundance of *Oedocephalum* was increased.

Fig 4. This is the Fig 4 Title. Bacterial and fungal community diversity in maize rhizosphere soil. CK: non-inoculated, J101CS: application with clear supernatant, J101BS: inoculated with the bacteria, J101FL: inoculated with the mixture of supernatant and bacteria.

Linkages between maize growth and soil properties

To determine the relationship between soil properties and maize growth, firstly we use Detrended correspondence analysis (DCA) to conduct on the growth indicators of maize and soil factors, and the maximum

gradient length of the two sort axes was 0.24, which is less than 3.0. Therefore, Redundancy analysis (RDA) was used to analyze the relationship between maize growth indicators and soil properties. The RDA analysis revealed that the contribution of the first axis to maize growth is 85.16%, and the total contribution for maize growth reached 97.66%. As shown in Fig. 5. The height, stem thickness, dry weight, SOD and POD activity in aboveground biomass and roots of maize are positively associated with the residual fraction of Pb in maize rhizosphere soil. Meanwhile, the maize’s height, stem thickness and fresh weight, SOD and POD activity in the roots of maize also are positively associated with the sub-stable fraction of soil Pb, such as strong organic-bound, iron-manganese oxide-bound and humic acid-bound fractions of Pb. However, the content of MDA and Pb in maize plants, and SOD, POD activity in the leaves of maize have a positive association with the ion-exchange fractions of Pb in maize rhizosphere soil. I There was a positive association between soil ACP and soil AP content. The content of AP in soil was positive association with both the P content in roots of maize, as well as the nitrogen content in aboveground biomass and roots in maize.

Fig 5. This is the Fig 5 Title. RDA analysis of physiological indicators of maize and soil factors. Pb-WS: water-soluble forms, Pb-IE: Ion-exchange forms, Pb-CB: Carbonate-bound forms, Pb-IMO: iron-manganese oxide-bound forms, Pb-SO: strong organic forms, Pb-HAB: Humic acid-bound forms, Pb-R: residual forms, AP: available phosphorus, AN: alkaline hydrolysis nitrogen, ACP: acidic phosphatase, URE: urease activity, pH: pH value of soil, maize-SOD-l: SOD activity in leaves of maize, maize-SOD-r: SOD activity in roots of maize, maize-POD-l: POD activity in leaves of maize, maize-POD-r: POD activity in roots of maize, maize-MDA-l: MDA content in leaves of maize, maize-MDA-r: MDA content in roots of maize, maize-N-a: N content in aboveground biomass of maize, maize-N-r: N content in roots of maize, maize-P-a: P content in aboveground

biomass of maize, maize-P-r: P content in roots of maize, maize-Pb-a: Pb content in aboveground biomass of maize, maize-Pb-r: Pb content in roots of maize, Plant height: plant height of maize, Stem thickness: stem thickness of maize, Fresh-weight-a: Fresh weight in aboveground biomass of maize, Fresh-weight-r: Fresh weight in roots of maize, Dry-weight-a: Dry weight in aboveground biomass of maize, Dry -weight-r: Dry weight in roots of maize.

Discussion

Mechanism of P solubilization by phosphate solubilizing bacteria

Previous studies have shown that PSB can significantly increase the soluble P in soil [38, 39]. In this study, the PSB *P. rwandensis* not only ~~can dissolve~~ $Ca_3(PO_4)_2$ but also ~~showed a certain tolerance to~~ Pb (Fig. 1 A-B). We found that the amount of bioavailable P ~~was~~ increased in the culture medium, and the pH of medium decreased. The secretion of organic acids, such as oxalic acid, tartaric acid, lactic acid and acetic acid, was detected in the culture medium (Fig. 1C), indicating that the organic acids by PSB *P. rwandensis* are an important mechanism to facilitate the solubilization of P from mineral phosphates through acidification and mental chelation. Generally, PSB release organic acid metabolites during biochemical processes, and it can reduce the environmental pH [40-43]. And these organic acids contain multiple carboxyl or hydroxyl groups, which can chelate Ca^{2+} cations bound and convert phosphate ions into soluble phosphates, and results in releasing the amount of bioavailable P [44-46]. The $Ca_3(PO_4)_2$ solubilization capacity of bacteria isolated from mineral phosphates was correlated with the organic acids secreted by the strains of cowpea nodules [47], which is in agreement with our findings. However, some studies PSB *Bacillus thuringiensis* and *P. ananatis* inoculated soil increased pH in lettuce rhizosphere soil should be the change of soil microbial community and a greater excreted quantity of HCO_3^- (OH^-) than H^+ by root due to the greater absorption of anions (PO_4^{3-}) than cation [48]. In addition, PSB *P. rwandensis* showed a certain ability

of tolerance to Pb^{2+} (Fig. 1B). It may be that the bacterial *Pantoea* may detoxify heavy metals Pb, cadmium, zinc, and mercury by regulating ATPases involved in metal transport and the copper-transporting P-type ATPase gene copA [49].

Impacts on growth of maize

Inoculated PSB may improve plant development and protect plants against heavy metal through various mechanisms, such as phosphate solubilization, IAA, endogenous oxidants and immobilization of heavy metal [49]. In heavy metal-contaminated soil, plants may improve endogenous oxidants (ROS) to alleviate the stress of free radicals inside cells, which was produced under the heavy metal stress [50]. In this study, compared to the uninoculated treatment, the inoculated PSB *P. rwandensis* significantly increased the activities of SOD and POD in maize roots (Fig. 2B-C), and significantly reduced the MDA content in leaves and roots of maize (Fig. 2D), indicating that the increase of activity of SOD and POD enhances the removal of superoxide anion radicals (O^{2-}) from maize roots and peroxidase further detoxifies them. This is consistent with [51] and [50], who demonstrated that inoculated *Enterobacter bugandensis, Serratia marcescens* and *Bacillus anthracis* in Cd, Pb and Cr contaminated soil can promote SOD and POD of *water spinach* and *Sesbania sesban*. Reactive oxygen species (ROS) are produced more frequently in plant cells as heavy metal content increase in plant, which damages cellular constituents. The ROS accumulation would further stimulate the production of MDA within the cells, therefore, MDA content in cells is significantly positively correlated with the heavy metal accumulation in plants [52, 53]. The soil inoculation experiment found that Pb accumulation in maize biomass was reduced by inoculation of *P. rwandensis* (Fig. 2A), which may result in a decrease of MDA in maize.

To some extent, microorganisms in inoculants may promote plant development and nutrient uptake by improving soil properties. In this study, we found that inoculated *P. rwandensis* significantly increased the height,

stem thickness, root and leave biomass in maize, while such effects were more significant in soil inoculated with only *P. rwandensis* (Table 1). The IAA with the value of 34.14 mg·L^{-1} secreted by PSB *P. rwandensis* was detected, and the increase of this compound in maize rhizosphere soil promoted maize growth, especially root biomass (Fig. S2). Microorganisms producing IAA can stimulate cell elongation or affect cell division to increase root growth and lengthen roots in plants, which is attributed to a larger roots surface area and allows plants to obtain more nutrients from the soil [54]. The genus *Pantoea* has the genes ipdC and amiE, which code for the production of IAA, and have two operons (speAB and speDE), which have been linked to the formation of lateral roots and production of spermidine to relieve oxidative stress [55].

Inoculated *P. rwandensis* significantly increased the soil available P contents, and increased maize's N uptake (Fig. 3A-B), which may contribute to promoting plant growth. In this study, the PSB *P. rwandensis* could secrete oxalic acid, tartaric acid, lactic acid and acetic acid (Fig. 1C), which is attributed to reducing pH of the soil (Table 3). Decreased soil pH dissolved the insoluble inorganic P and then increased the amount of bioavailable P in the soil [51]. Meanwhile, the inoculation of PSB increased soil ACP activity (Table 3). The ACP could hydrolyze phosphodiester and anhydrides in soil to release, inorganic phosphorus which resulted in the increase of bioavailable P in soil [56-58]. In this study, inoculation of *P. rwandensis* leaded to an increase of soil available P nutrients, since P is one of the most essential plant nutrients and it widely involved in many key physiological and metabolic processes in plants, including cell division and development, energy transport, signal transduction, macromolecular biosynthesis, photosynthesis, and respiration [59]. Indeed, it was found that there was a significant increase in N uptake by maize, although there was no significant change in the available N content of the soil (Fig. 3A). The improved P supply by PSB inoculation may regulate ATP hydrolysis to release energy, which to affect metabolic processes such as protein and amino acid synthesis, so that the N content of the plant

increases [60].

It has been reported that Pb in soil was converted into residual Pb fractions, for example, Pb-phosphates compounds, its phytoavailbilty significantly reduced [8]. In this study, the PSB *P. rwandensis* could efficiently use $Ca_3(PO_4)$ as the sole P source and it increase the water-soluble phosphate amount in culture media. In soil inoculation experiment showed that available P content in maize rhizosphere soil was increased, as well as the residual Pb fractions in Pb contaminated soil were significantly increased by the inoculation of *P. rwandensis*, especially the fraction of residual Pb content in maize rhizosphere soil inoculated by bacteria solution increased significantly by 89.81%, higher than that of the other treatments (Table 2). These results confirmed that the inoculation of PSB *P. rwandensis* promoted Pb immobilization and decrease the phytoavailability of Pb, which may attribute to a decrease in Pb accumulation of maize biomass. This is consistent with the work of Liu, who demonstrated that PSB *Pantoe sp.* effectively immobilized Pb in lettuce rhizosphere soil [48]. Because of the P-releasing activity of PSB, the increase of solubilizing P from mineral phosphates could facilitate the formation of residual Pb-phosphorate compounds in the soil. The plant height, stem thickness and biomass of maize were positively associated with residual Pb fraction in the soil. (Fig. 5). These findings are consistent with the commonly viewpoint that the increase of soil available P further facilitated the formation of insoluble residual Pb compounds. But the formation process of Pb-phosphorate compound fraction would be need of further study.

Impact on soil microbial community diversity

Microbial distributions can be markedly changed by inoculating functional microbes, but the disturbance trend of the microbial community was inconclusive [61, 62]. In this study, Inoculation of *P. rwandensis* had no significant effect on Chao and Simpson indices of soil bacterial communities, but application of clear supernatant with secretion of *P. rwandensis* and inoculated *P. rwandensis* increased Chao index and decreased Simpson index

of soil fungal communities in maize rhizosphere soil (Table 4). This result indicated that inoculation of functional microbes' PSB promoted the biodiversity of the fungal community and affect the biodiversity of the fungal community more significantly than that of bacterial communities. This phenomenon may be related to the competition for resources between PSB and soil microbial groups, making it more competitive by providing attachment points and inhibiting bacterial growth. In addition, the inoculated *P. rwandensis* is resistant to heavy metal Pb (Fig. 1B), so inoculation of it reduced the abundance of the dominant phylum *Ascomycota* in the fungal community and increased the dominance of the sub-dominant genus *Oedocephalum*, which improved the biodiversity of the fungal community by reducing the competitiveness of the dominant phylum and genera of fungi and providing resources for the propagation of other fungi.

The application of clear supernatant with the secretion of *P. rwandensis* and inoculated *P. rwandensis* increased the relative abundance of the dominant *Proteobacteria* in bacterial community, and the secretion of *P. rwandensis* promoted the abundance genus of *Sphingomonas*. This result was consistent with the studies of [63]. who the inoculation of *Pseudoalteromonas agarivorans* promoted the genus of *Proteobacteria*, *Sphingomonas* in rhizosphere soil of *Brassica chinensis* L. in Pb^{2+}contaminated soil. The genus *Proteobacteria* had a fixation to heavy metal or growth promotion to plants, and the genus *Sphingomonas* had high tolerance to heavy metal by absorption, precipitation and oxidation-reduction. These microorganisms are dominant genera for metal immobilization and plants growth promotion [63]. In this study, the increase of *Proteobacteria* and *Sphingomonas* genus indicated that inoculation of PSB promoted the relative abundance of microorganisms with heavy metal Pb^{2+} resistance. However, the dominance of the fungal *Mortierella* was increased after the application of clear supernatant with the secretion of *P. rwandensis* (Fig. 4D). some studies showed that the fungal *Mortierella* had a

high tolerance and adsorption capacity for heavy metals and can effectively reduce the bioavailability of Pb [64, 65].

Conclusion

The current study demonstrated that (i) the Pb-resistant *P. rwandensis* promoted the solubilization of insoluble $Ca_3(PO_4)_2$ and showed a certain tolerance to Pb^{2+}. Its main solubilization mechanism of mineral P is pH reduction by secreting various organic; (ii) inoculations with *P. rwandensis* significantly increased the amount of residual Pb fraction in maize rhizosphere soil, indicating that the Pb-resistant PSB helped transform the mobilization fraction Pb into immobilization fraction Pb in soil. *P. rwandensis* inoculation promote the biodiversity of the fungal community in soil, and significantly increases the abundance of heavy metal-tolerant microorganisms *Proteobacteria*, *Mortierella*, and *Sphingomonas* in maize rhizosphere soil; (iii) *P. rwandensis* inoculation promote maize growth, and significantly reduced the Pb accumulation in maize biomass and inhibited the transfer of Pb from the roots to the aboveground parts. Additionally, *P. rwandensis* effectively increased antioxidant enzyme activity in maize roots and decreased MDA content in maize, resulting in increase of maize tolerance to heavy metal Pb. However, it should be noted that further research is required to investigate the process of Pb species transformation in soil systems.

Acknowledgements

This work is funded by National Natural Science Foundation of China (42167009), Joint Fund Project of Yunnan Provincial Universities (2018FH001-004), Scientific Research Fund Project of Yunnan Provincial Department of Education (2022Y712, 2023Y0874), The International Joint Innovation Team for Yunnan Plateau Lakes and Laurentian Great Lakes (funded by Yunnan Provincial Education Department), College Students' Innovation and Entrepreneurship project (202111393070).

References

1. Gao YN, Wu P, Jeyakumar P, Bolan N, Wang HL, Gao B, et al. Biochar as a potential strategy for remediation of contaminated mining soils: Mechanisms, applications, and future perspectives. Journal of Environmental Management. 2022. doi: 10.1016/j.jenvman.2022.114973.
2. Panagos P, Van Liedekerke M, Yigini Y, Montanarella L. Contaminated Sites in Europe: Review of the Current Situation Based on Data Collected through a European Network. Journal of Environmental and Public Health,2013,(2013-6-16). 2013;2013:158764.
3. Cetin M, Aljama AMO, Alrabiti OBM, Adiguzel F, Sevik H, Zeren Cetin I. Determination and Mapping of Regional Change of Pb and Cr Pollution in Ankara City Center. Water, Air, & Soil Pollution. 2022. doi: 10.1007/s11270-022-05638-1.
4. Jiang Q, He YM, Wu YL, Dian B, Zhang JL, Li TG, et al. Solidification/stabilization of soil heavy metals by alkaline industrial wastes: A critical review. Environmental Pollution. 2022. doi: 10.1016/j.envpol.2022.120094.
5. Massimiliano P, Alessio P, Serenella M, Rosita C, Valeria Marina N, Maria Antonietta Z. Metal Toxicity and Speciation: A Review. Current Medicinal Chemistry. 2021. doi: 10.2174/0929867328666210324161205.
6. Xu JC, Huang LM, Chen C, Wang J, Long XX. Effective lead immobilization by phosphate rock solubilization mediated by phosphate rock amendment and phosphate solubilizing bacteria. Chemosphere. 2019. doi: 10.1016/j.chemosphere.2019.124540.
7. Park JH, Bolan N, Megharaj M, Naidu R. Isolation of phosphate solubilizing bacteria and their potential for lead immobilization in soil. Journal of Hazardous Materials. 2010. doi: 10.1016/j.jhazmat.2010.09.095.
8. Zeng GM, Wan J, Huang DL, Hu LA, Huang C, Cheng M, et al. Precipitation, adsorption and rhizosphere effect: the mechanisms for Phosphate-induced Pb immobilization in soils-A review. Journal of Hazardous Materials. 2017. doi: 10.1016/j.jhazmat.2017.05.038.
9. Kilgour DW, Moseley RB, Barnett MO, Savage KS, Jardine PM. Potential Negative Consequences of doi: 10.2134/jeq2007.0409.
10. Weber JS, Goyne KW, Luxton TP, Thompson AL. Phosphate Treatment of Lead-Contaminated Soil: Effects on Water Quality, Plant Uptake, and Lead Speciation. Journal of Environmental Quality. 2015. doi: 10.2134/jeq2014.10.0447.
11. Chen HM, Zhang JW, Tang LY, Su M, Tian D, Zhang L, et al. Enhanced Pb immobilization via the combination of biochar and phosphate solubilizing bacteria. Environment International. 2019. doi: 10.1016/j.envint.2019.03.068.
12. Huang GY, Gao RL, You JW, Zhu J, Fu QL, Hu HQ. Oxalic acid activated phosphate rock and bone meal to immobilize Cu and Pb in mine soils. Ecotoxicology and Environmental Safety. 2019. doi: 10.1016/j.ecoenv.2019.02.076.
13. Dasila H, Sah VK, Jaggi V, Kumar A, Tewari L, Taj G, et al. Cold-tolerant phosphate-solubilizing Pseudomonas strains promote wheat growth and yield by improving soil phosphorous (P) nutrition status. Frontiers in Microbiology. 2023. doi: 10.3389/fmicb.2023.1135693.
14. Azeem M, Hassan TU, Tahir MI, Ali A, Jeyasundar PGSA, Hussain Q, et al. Tea leaves biochar as a carrier of Bacillus cereus improves the soil function and crop productivity. Applied Soil Ecology. 2021. doi: 10.1016/j.apsoil.2020.103732.
15. Guo JK, Muhammad H, Lv X, Wei T, Ren XH, Jia HL, et al. Prospects and applications of plant growth

promoting rhizobacteria to mitigate soil metal contamination: A review. Chemosphere. 2020. doi: 10.1016/j.chemosphere.2020.125823.

16. Dong RS, Zhang J, Huan HF, Bai CJ, Chen ZJ, Liu GD. High Salt Tolerance of a Bradyrhizobium Strain and Its Promotion of the Growth of Stylosanthes guianensis. International Journal of Molecular Sciences. 2017. doi: 10.3390/ijms18081625.
17. Mariajosé C, Liliana G, Marlene G, Daniela C, Francisco A. Screening for indole-3-acetic acid synthesis and 1-aminocyclopropane-carboxylate deaminase activity in soil yeasts from Chile uncovers Solicoccozyma aeria as an effective plant growth promoter. Plant and Soil. 2023. doi: 10.1007/s11104-023-05906-x.
18. Sun SL, Yang WL, Fang WW, Zhao YX, Guo L, Dai YJ. The Plant Growth-Promoting Rhizobacterium Variovorax boronicumulans CGMCC 4969 Regulates the Level of Indole-3-Acetic Acid Synthesized from Indole-3-Acetonitrile. Applied and Environmental Microbiology. 2018. doi: 10.1128/aem.00298-18.
19. Horstmann JL, Dias MP, Ortolan F, Medina-Silva R, Astarita LV, Santarém ER. Streptomyces sp. CLV45 from Fabaceae rhizosphere benefits growth of soybean plants. Brazilian Journal of Microbiology. 2020. doi: 10.1007/s42770-020-00301-5.
20. Hanaka A, Ozimek E, Majewska M, Rysiak A, Jaroszuk-Ściseł J. Physiological Diversity of Spitsbergen Soil Microbial Communities Suggests Their Potential as Plant Growth-Promoting Bacteria. International Journal of Molecular Sciences. 2019. doi: 10.3390/ijms20051207.
21. Ratnaningsih HR, Noviana Z, Dewi TK, Loekito S, Wiyono S, Gafur A, et al. IAA and ACC deaminase producing-bacteria isolated from the rhizosphere of pineapple plants grown under different abiotic and biotic stresses. Heliyon. 2023. doi: 10.1016/j.heliyon.2023.e16306.
22. Aberathna AAAU, Satharasinghe DA, Jayasooriya AP, Jinadasa RN, Manopriya S, Jayaweera BPA, et al. Increasing the Bioavailability of Phosphate by Using Microorganisms. International Journal of Agronomy. 2022. doi: 10.1155/2022/4305501.
23. Jiang YF, Ge F, Li F, Zhang DY, Deng SQ, Tian J. Intracellular Metabolomics Switching Alters Extracellular Acid Production and Insoluble Phosphate Solubilization Behavior in Penicillium oxalicum. Metabolites. 2020. doi: 10.3390/metabo10110441.
24. Chen SY, Yang D, Wei YF, He LZ, Li ZJ, Yang SD. Changes in Soil Phosphorus Availability and Microbial Community Structures in Rhizospheres of Oilseed Rapes Induced by Intercropping with White Lupins. Microorganisms. 2023. doi: 10.3390/microorganisms11020326.
25. Basak BB. Phosphorus Release by Low Molecular Weight Organic Acids from Low-Grade Indian Rock Phosphate. Waste and Biomass Valorization. 2018. doi: 10.1007/s12649-018-0361-3.
26. Weissengruber L, Möller K, Puschenreiter M, Friedel JK. Long-term soil accumulation of potentially toxic elements and selected organic pollutants through application of recycled phosphorus fertilizers for organic farming conditions. Nutrient Cycling in Agroecosystems. 2018. doi: 10.1007/s10705-018-9907-9.
27. Li H, Li DH, Xiao TF, He LB, Ning ZP, Sun JL, et al. Geochemistry and environmental effect of cadmium in the super-large Jinding Pb-Zn deposit, Yunnan Province, China. Chinese Journal of Geochemistry. 2008. doi: 10.1007/s11631-008-0021-6.
28. Koch M, Kruse J, Eichler-Löbermann B, Zimmer D, Willbold S, Leinweber P, et al. Phosphorus stocks and speciation in soil profiles of a long-term fertilizer experiment: Evidence from sequential fractionation, P K -edge XANES, and 31 P NMR spectroscopy. Geoderma. 2018. doi: 10.1016/j.geoderma.2017.12.003.
29. Liu J, Qi W, Li Q, Wang SG, Song C, Yuan XZ. Exogenous phosphorus-solubilizing bacteria changed the rhizosphere microbial community indirectly. 3 Biotech. 2020. doi: 10.1007/s13205-020-2099-4.

30. Wang ZH, Zhang HH, Liu L, Li SJ, Xie JF, Xue X, et al. Screening of phosphate-solubilizing bacteria and their abilities of phosphorus solubilization and wheat growth promotion. BMC Microbiology. 2022. doi: 10.1186/s12866-022-02715-7.
31. Qi WY, Chen H, Wang Z, Xing SF, Song C, Yan Z, et al. Biochar-immobilized Bacillus megaterium enhances Cd immobilization in soil and promotes Brassica chinensis growth. Journal of Hazardous Materials. 2023. doi: 10.1016/j.jhazmat.2023.131921.
32. Li Z, Su M, Duan X, Tian D, Yang M, Guo J, et al. Induced biotransformation of lead (II) by Enterobacter sp. in SO_4-PO_4-Cl solution. Journal of Hazardous Materials. 2018. doi: 10.1016/j.jhazmat.2018.06.032.
33. Zhang XS. Analysis of the Factors Affecting the Available P Content in the Fermentation Liquid of P Bacteria Determined by Mo-Sb Colorimetry. Journal of Anhui Agricultural Sciences. 2008.
34. Su M, Han FY, Wu YL, Yan ZP, Lv ZS, Tian D, et al. Effects of phosphate-solubilizing bacteria on phosphorous release and sorption on montmorillonite. Applied Clay Science. 2019. doi: 10.1016/j.clay.2019.105227.
35. Khan N, Bano A, Rahman MA, Guo J, Kang Z, Babar MA. Comparative Physiological and Metabolic Analysis Reveals a Complex Mechanism Involved in Drought Tolerance in Chickpea (Cicer arietinum L.) Induced by PGPR and PGRs. Scientific Reports. 2019. doi: 10.1038/s41598-019-38702-8.
36. Biswas JK, Banerjee A, Rai M, Naidu R, Biswas B, Vithanage M, et al. Potential application of selected metal resistant phosphate solubilizing bacteria isolated from the gut of earthworm (Metaphire posthuma) in plant growth promotion. Geoderma. 2018. doi: 10.1016/j.geoderma.2018.05.034.
37. Lu RK. Soil agrochemical analysis method. China Agricultural Science and Technology Press.2000.
38. Isidro BM, Felipe RP, Lady MC, Angelica YG, Antonio MMS, German EB. Inoculation of phosphate-solubilizing bacteria improves soil phosphorus mobilization and maize productivity. Nutrient Cycling in Agroecosystems. 2023. doi: 10.1007/s10705-023-10268-y.
39. Li HP, Han QQ, Liu QM, Gan YN, Rensing C, Rivera WL, et al. Roles of phosphate-solubilizing bacteria in mediating soil legacy phosphorus availability. Microbiological Research. 2023. doi: 10.1016/j.micres.2023.127375.
40. Ma Y, Rajkumar M, Freitas H. Inoculation of plant growth promoting bacterium Achromobacter xylosoxidans strain Ax10 for the improvement of copper phytoextraction by Brassica juncea. Journal of Environmental Management. 2008. doi: 10.1016/j.jenvman.2008.01.014.
41. Sugiura H, Sugihara S, Kamiya T, Artigas Ramirez MD, Miyatake M, Fujiwara T, et al. Sulfur application enhances secretion of organic acids by soybean roots and solubilization of phosphorus in rhizosphere. Soil Science and Plant Nutrition. 2021. doi: 10.1080/00380768.2021.1919011.
42. Chen WM, Yang F, Zhang L, Wang JM. Organic Acid Secretion and Phosphate Solubilizing Efficiency ofPseudomonas sp. PSB12: Effects of Phosphorus Forms and Carbon Sources. Geomicrobiology Journal. 2016. doi: 10.1080/01490451.2015.1123329.
43. Yang TY, Li LB, Tian J, Zhang SS, Wu ZQ. [Relationships between phosphate solubilizing ability, pH, and organic acids of Penicillium oxalicum YTY and its mutants]. Ying yong sheng tai xue bao = The journal of applied ecology. 2021. doi: 10.13287/j.1001-9332.202112.038.
44. Xue SW, Wang YW, Bo WT, Wan KJ, Miao ZY. Calcium-doped magnetic humic acid nano particles for the efficient removal of heavy metals from wastewater: the role of Ca. Environmental Technology. 2023. doi: 10.1080/09593330.2023.2213832.
45. Harrold SA, Tabatabai MA. Release of Inorganic Phosphorus from Soils by Low- Molecular- Weight

Organic Acids. Communications in Soil Science and Plant Analysis. 2006. doi: 10.1080/00103620600623558.

46. Yeh TY, Lin CL, Lin CF, Chen CC. Chelator-enhanced phytoextraction of copper and zinc by sunflower, Chinese cabbage, cattails and reeds. International Journal of Environmental Science and Technology. 2014. doi: 10.1007/s13762-014-0592-1.
47. Marra LM, de Oliveira-Longatti SM, Soares CRFS, Olivares FL, Moreira FMdS. The Amount of Phosphate Solubilization Depends on the Strain, C-Source, Organic Acids and Type of Phosphate. Geomicrobiology Journal. 2019. doi: 10.1080/01490451.2018.1542469.
48. Liu XY, Chen CY, Wang J, Zou SH, Long XX. Phosphorus solubilizing bacteria Bacillus thuringiensis and Pantoea ananatis simultaneously promote soil inorganic phosphate dissolution and soil Pb immobilization. Rhizosphere. 2021. doi: 10.1016/j.rhisph.2021.100448.
49. Luziatelli F, Ficca AG, Cardarelli M, Melini F, Cavalieri A, Ruzzi M. Genome Sequencing of Pantoea agglomerans C1 Provides Insights into Molecular and Genetic Mechanisms of Plant Growth-Promotion and Tolerance to Heavy Metals. Microorganisms. 2020. doi: 10.3390/microorganisms8020153.
50. Ali J, Ali F, Ahmad I, Rafique M, Munis MFH, Hassan SW, et al. Mechanistic elucidation of germination potential and growth of Sesbania sesban seedlings with Bacillus anthracis PM21 under heavy metals stress: An in vitro study. Ecotoxicology and Environmental Safety. 2020. doi: 10.1016/j.ecoenv.2020.111769.
51. Wang X, Cai D, Ji M, Chen Z, Yao L, Han H. Isolation of heavy metal-immobilizing and plant growth-promoting bacteria and their potential in reducing Cd and Pb uptake in water spinach. Science of the Total Environment. 2022. doi: 10.1016/j.scitotenv.2022.153242.
52. Wu YH, Chen YH, Yi YH, Shen ZG. Responses to copper by the moss Plagiomnium cuspidatum: hydrogen peroxide accumulation and the antioxidant defense system. Chemosphere. 2008. doi: 10.1016/j.chemosphere.2008.10.059.
53. Li N, Sheng KY, Zheng QY, Hu DN, Zhang L, Wang JW, et al. Inoculation with phosphate-solubilizing bacteria alters microbial community and activates soil phosphorus supply to promote maize growth. Land Degradation & Development. 2022. doi: 10.1002/ldr.4494.
54. Ma Y, Wang Y, Shi XJ, Chen XP, Li ZL. [Mechanism and Application of Plant Growth-Promoting Bacteria in Heavy Metal Bioremediation]. Huan jing ke xue= Huanjing kexue. 2022. doi: 10.13227/j.hjkx.202112007.
55. Ejaz U, Khan SM, Khalid N, Ahmad Z, Jehangir S, Fatima Rizvi Z, et al. Detoxifying the heavy metals: a multipronged study of tolerance strategies against heavy metals toxicity in plants. Frontiers in Plant Science. 2023. doi: 10.3389/fpls.2023.1154571.
56. Priyanka U, Mehak G, Simarjeet Kaur S, Rakesh S, Sanjula S, Virender KS, et al. Genome wide association studies for acid phosphatase activity at varying phosphorous levels in Brassica juncea L. Frontiers in Plant Science. 2022. doi: 10.3389/fpls.2022.1056028.
57. Ma XF, Li HG, Zhang JL, Shen JB. Spatiotemporal pattern of acid phosphatase activity in soils cultivated with maize sensing to phosphorus-rich patches. Frontiers in Plant Science. 2021. doi: 10.3389/fpls.2021.650436.
58. An XX, Liu JY, Liu XS, Ma CH, Zhang QB. Optimizing Phosphorus Application Rate and the Mixed Inoculation of Arbuscular Mycorrhizal Fungi and Phosphate-Solubilizing Bacteria Can Improve the Phosphatase Activity and Organic Acid Content in Alfalfa Soil. Sustainability. 2022. doi: 10.3390/su141811342.
59. Fu YF, Yang XY, Zhang ZW, Yuan S. Synergistic effects of nitrogen metabolites on auxin regulating plant

growth and development. Frontiers in Plant Science. 2022. doi: 10.3389/fpls.2022.1098787.

60. Lai YH, Peng MY, Rao RY, Chen WS, Wei-Tao Huang WT, Ye X, et al. An Integrated Analysis of Metabolome, Transcriptome, and Physiology Revealed the Molecular and Physiological Response of Citrus sinensis Roots to Prolonged Nitrogen Deficiency. Plants. 2023. doi: 10.3390/plants12142680.
61. Galaviz C, Lopez BR, de-Bashan LE, Hirsch AM, Maymon M, Bashan Y. Root growth improvement of mesquite seedlings and bacterial rhizosphere and soil community changes are induced by inoculation with plant growth-promoting bacteria and promote restoration of eroded desert soil. Land Degradation & Development. 2018. doi: 10.1002/ldr.2904.
62. Bai BX, Yang X, Zhao QS, Liu RX, Ren JH. Inoculations with Pseudomonas fluorescens and Bacillus cereus affect the soil enzyme activity, growth and rhizosphere microbial diversity of Taxus chinensis var. mairei. Plant and Soil. 2020. doi: 10.1007/s11104-020-04660-8.
63. Cao RW, Zhang YL, Ju YH, Wang W, Zhao YQ, Liu N, et al. Exopolysaccharide-producing bacteria enhanced Pb immobilization and influenced the microbiome composition in rhizosphere soil of pakchoi (Brassica chinensis L.). Frontiers in Microbiology. 2023. doi: 10.3389/fmicb.2023.1117312.
64. Li XX, Zhang X, Cui ZJ. Combined bioremediation for lead in mine tailings by Solanum nigrum L. and indigenous fungi. Chemistry and Ecology. 2017. doi: 10.1080/02757540.2017.1394458.
65. Cui ZJ, Zhang X, Yang HH, Sun LQ. Bioremediation of heavy metal pollution utilizing composite microbial agent of Mucor circinelloides, Actinomucor sp. and Mortierella sp. Journal of Environmental Chemical Engineering. 2017. doi: 10.1016/j.jece.2017.07.021.

Supporting information

S1 Fig. This is the S1 Fig Title. Phylogenetic tree of strain based on 16S rDNA gene sequences.

S1 Fig. This is the S1 Fig Title. Root systems of maize under different treatment.

Figure1

Click here to access/download;Figure;Fig.1.tif

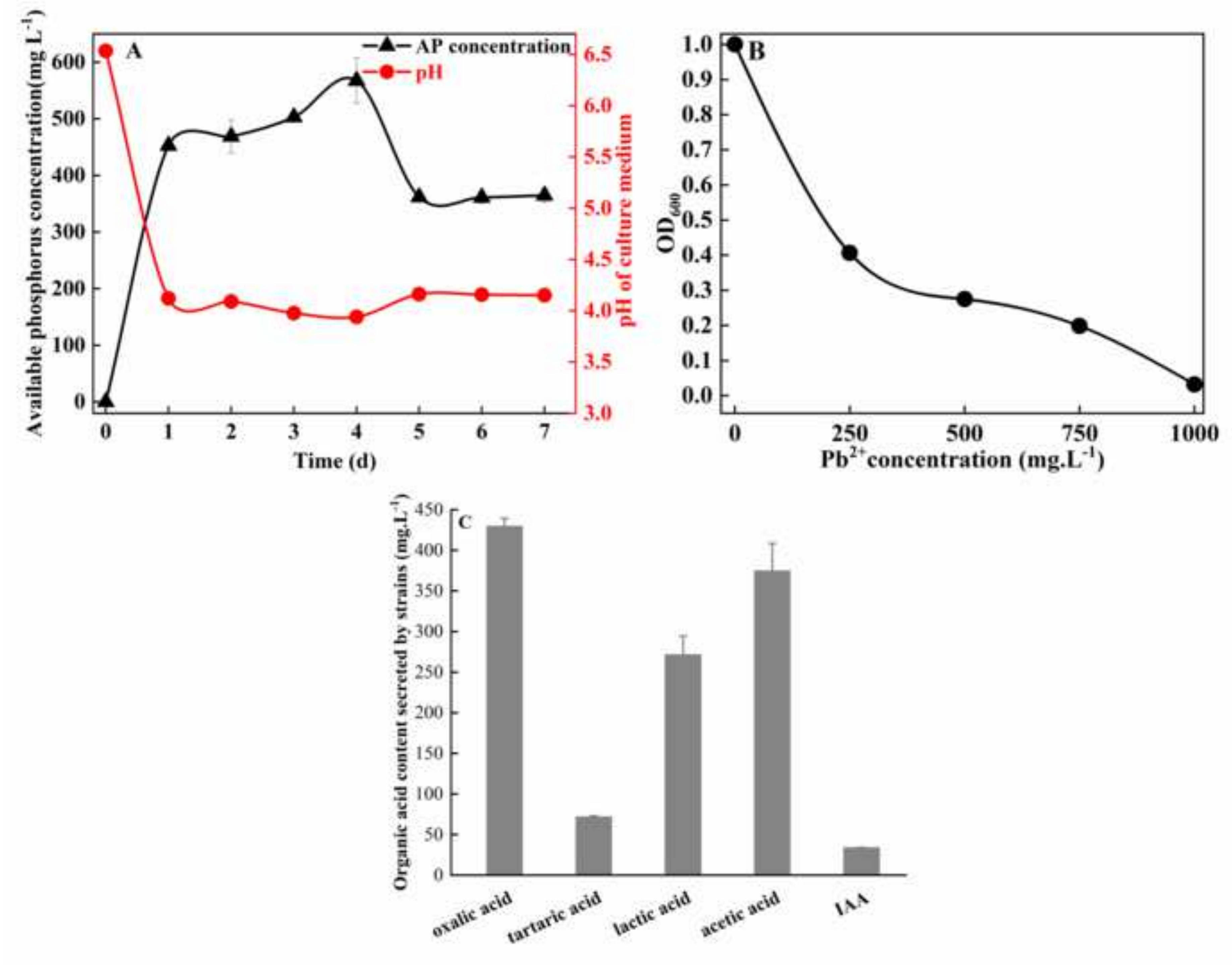

Figure2

Click here to access/download;Figure;Fig.2.tif

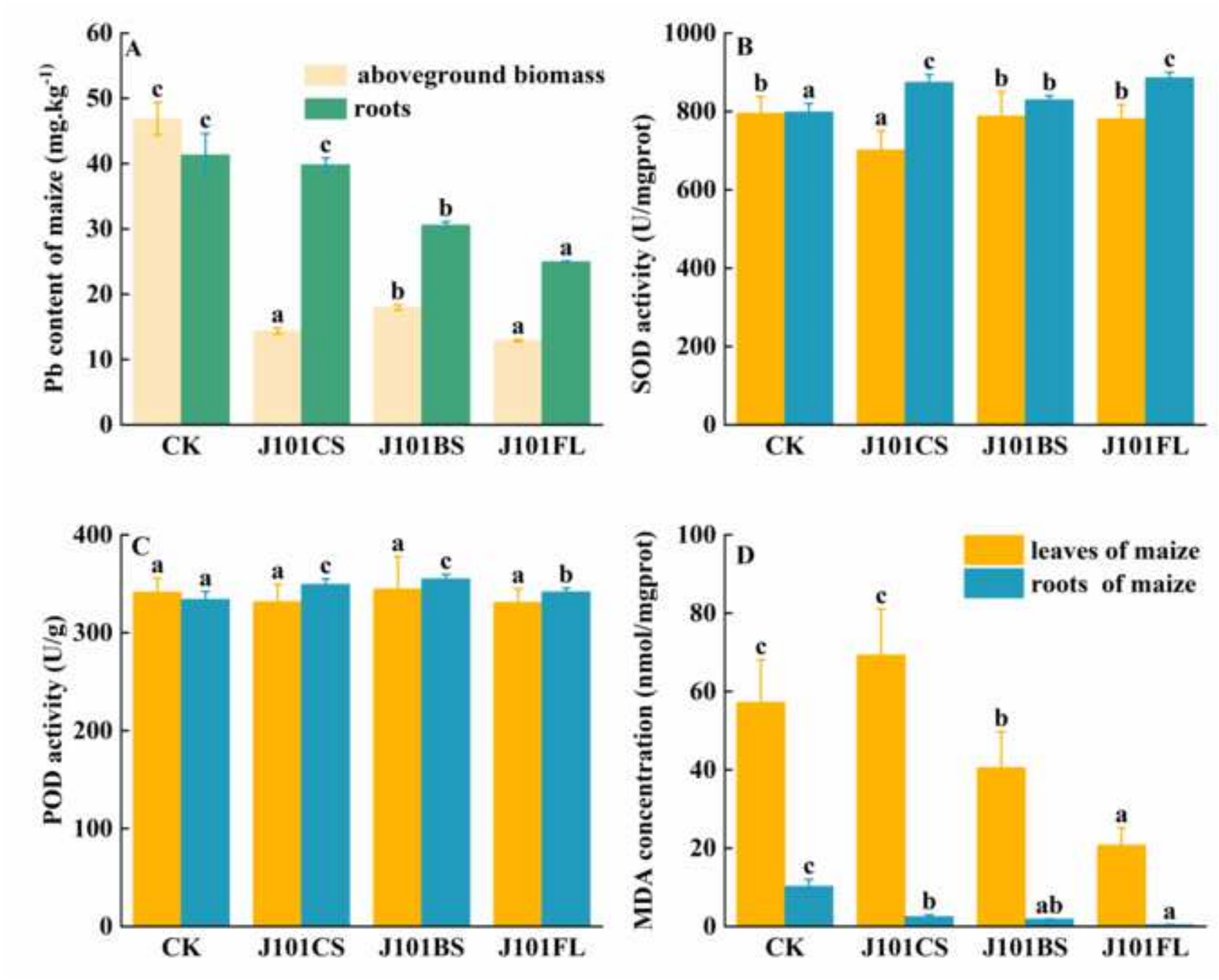

Figure3

Click here to access/download;Figure;Fig.3.tif

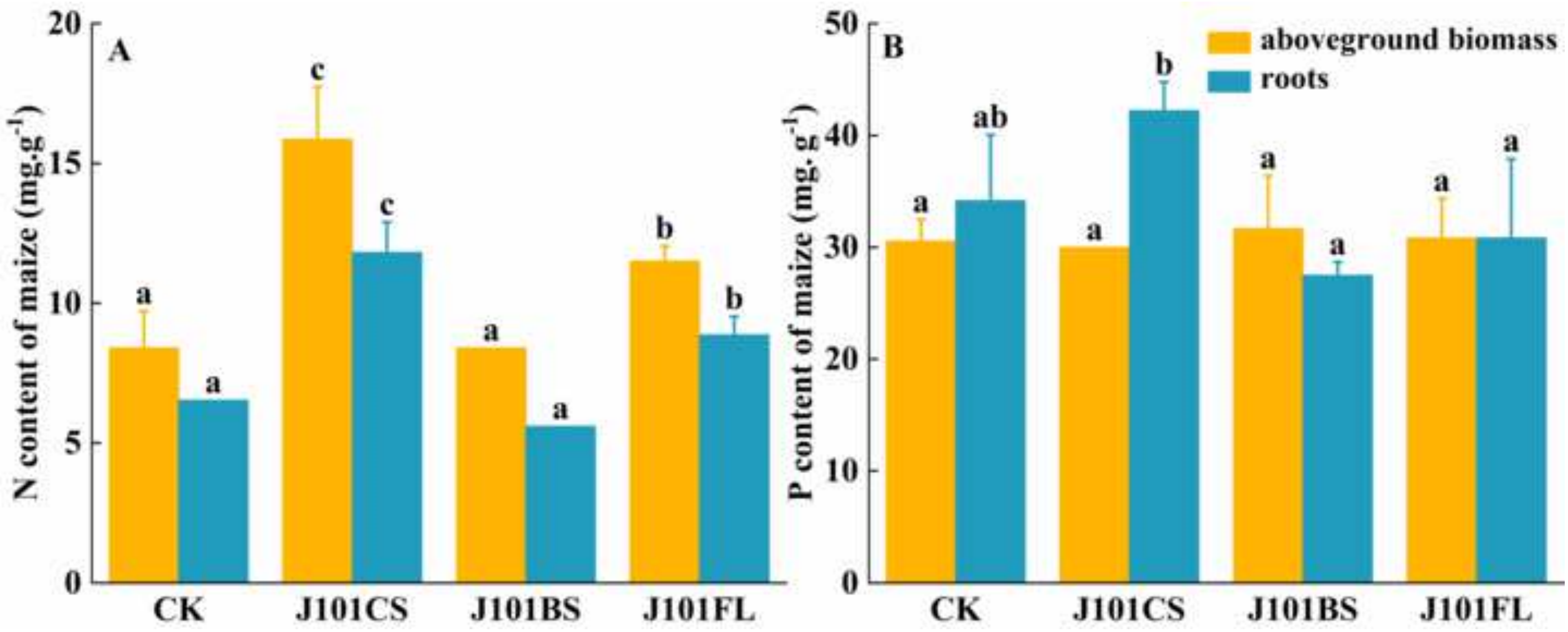

Figure4 [Click here to access/download;Figure;Fig.4.tif]

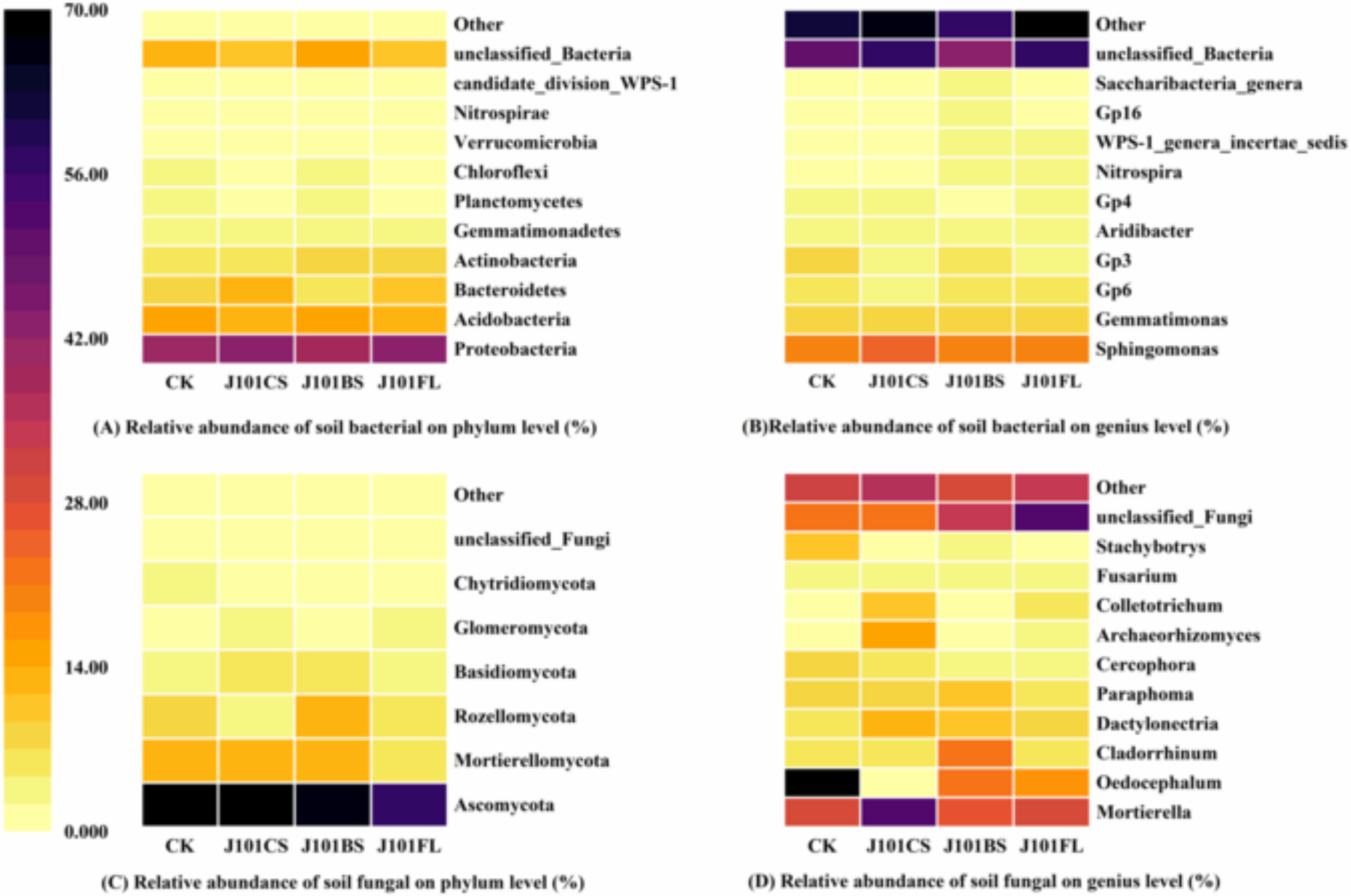

Figure5

Click here to access/download;Figure;Fig.5.tif

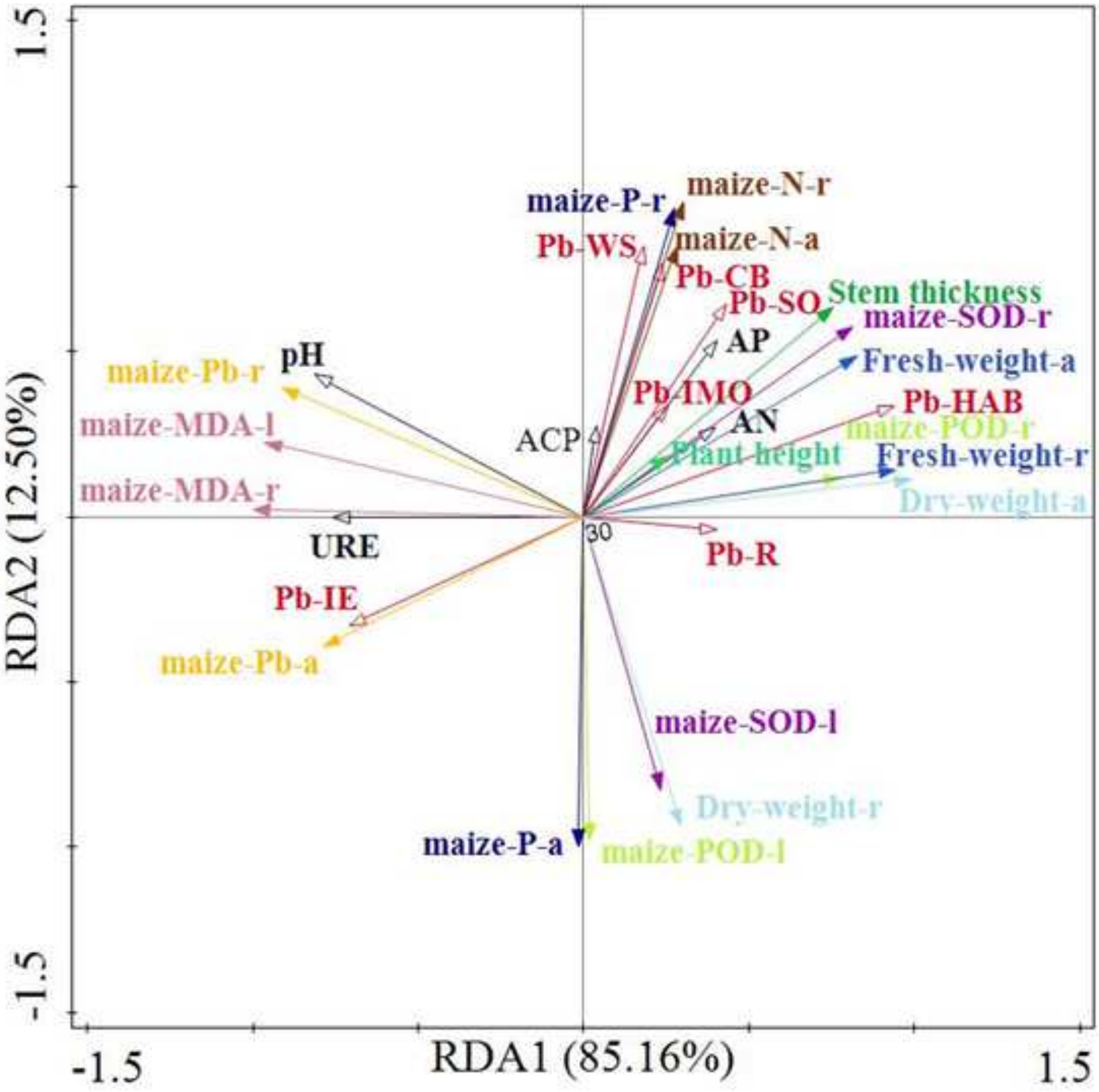

Figure. S1

Click here to access/download;Figure;Fig.S1.tif

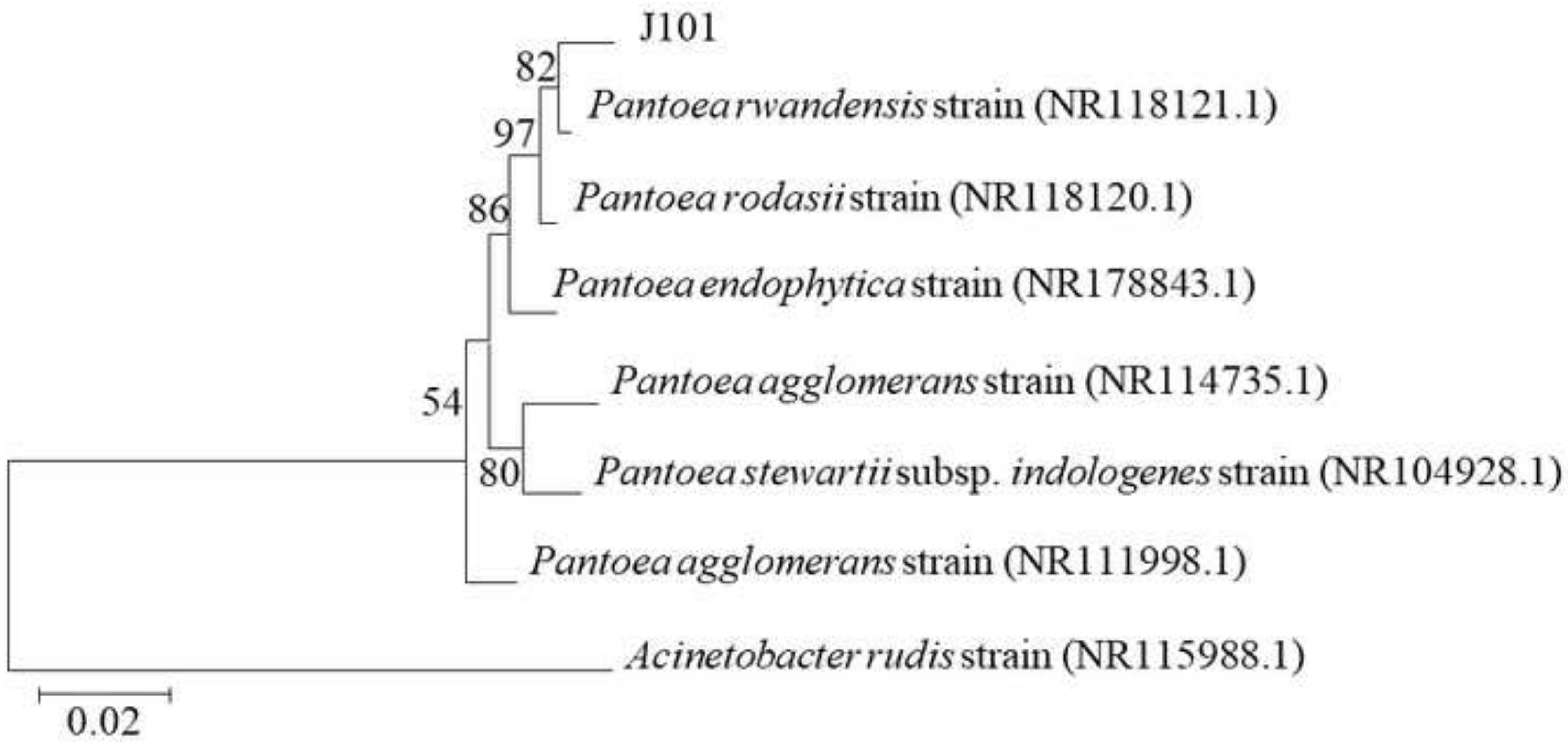

Figure. S2

Click here to access/download;Figure;Fig.S2.tif

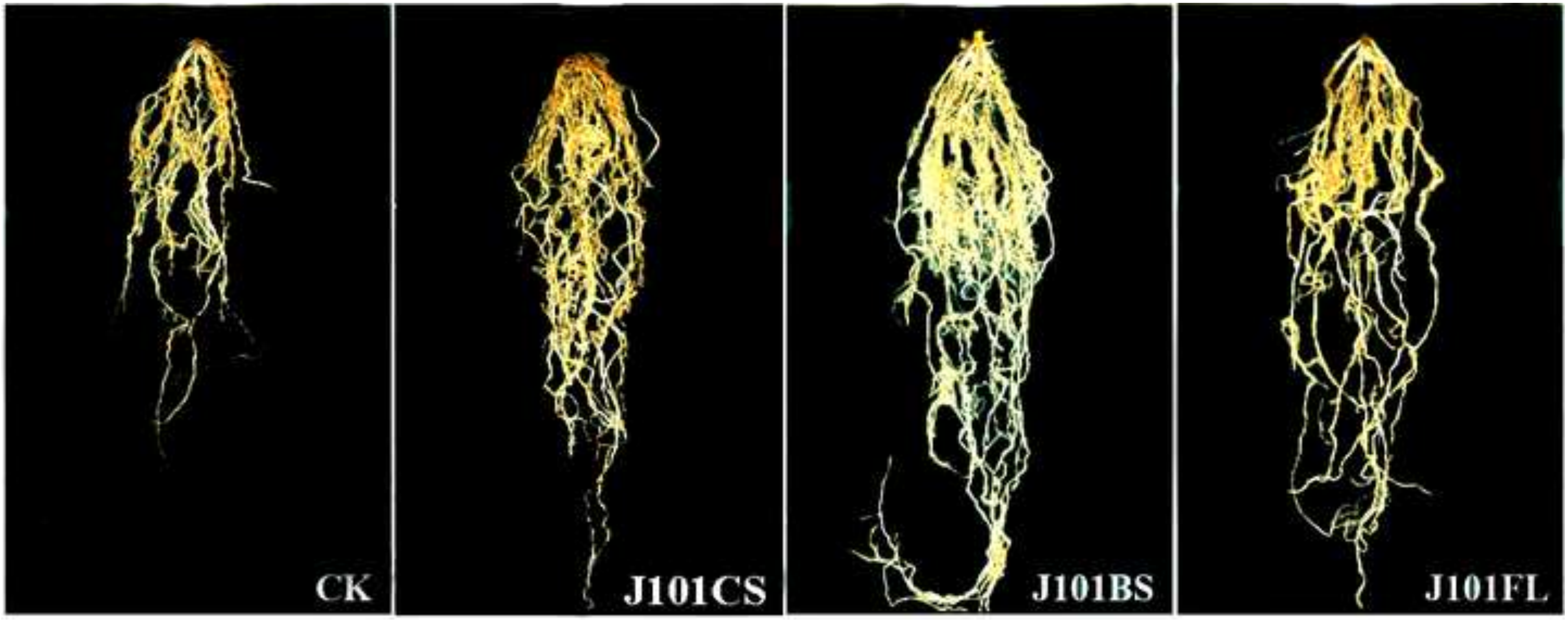

Table 1 Changes of biomass in maize.

	Plant height (cm)	Stem thickness (mm)	Fresh weight of aboveground (g)	Fresh weight of roots (g)	Dry weight of aboveground (g)	Dry weight of roots (g)
CK	26.30±1.64a	5.29±0.75a	3.28±0.84a	2.21±0.59a	0.57±0.14a	0.56±0.07a
J101CS	31.25±7.04b	6.86±1.35b	5.56±1.36b	6.08±1.22b	0.85±0.31b	0.47±0.05a
J101BS	31.83±7.34b	6.28±0.72b	5.78±0.90b	6.52±0.98b	0.88±0.08b	0.66±0.07b
J101FL	31.91±2.55b	7.00±1.04b	7.59±1.97c	9.16±2.79c	1.19±0.38c	0.66±0.12b

CK: non-inoculated, J101CS: application with clear supernatant, J101BS: inoculated with the bacteria, J101FL: inoculated with the mixture of supernatant and bacteria. One-way analysis of variance was performed followed by Duncan's multiple range test at a significance level of $p<0.05$. Different letters indicate statistically significant differences.

Table 2 The soil Pb forms in maize rhizosphere.

The forms of heavy metal Pb（$mg \cdot kg^{-1}$）	CK	J101CS	J101BS	J101FL
Water-soluble forms	3.32±0.01a	3.45±0.00c	3.39±0.00b	3.37±0.02b
Ion-exchange forms	168.41±2.05b	144.69±1.47a	144.14±0.63a	145.58±0.89a
Carbonate-bound forms	431.36±1.88a	700.98±11.91d	582.58±13.41c	535.61±24.37b
Humic acid-bound forms	7.44±0.09a	8.49±0.25b	8.18±0.18b	8.04±0.33b
Iron-manganese oxide-bound forms	1064.62±45.63a	1198.70±15.83b	1150.18±25.75b	1276.75±26.08c
Strong organic-bound forms	240.12±2.57a	335.52±21.88c	345.80±17.49c	283.76±22.11b
Residual forms	70.19±3.09a	104.98±0.38c	133.23±1.70d	99.45±2.43b

CK: non-inoculated; J101CS: application with clear supernatant, J101BS: inoculated with the bacteria, J101FL: inoculated with the mixture of supernatant and bacteria. One-way analysis of variance was performed followed by Duncan's multiple range test at a significance level of $p<0.05$. Different letters indicate statistically significant differences.

Table 3 Soil properties of maize rhizosphere.

	URE (mg.g^{-1}, 24^{-1})	ACP (mg.g^{-1}, 24h^{-1})	AP (mg.g^{-1})	AN (mg.g^{-1})	pH value
CK	18354.70±6379.09a	270.00±33.00a	14.40±1.68a	133.00±9.90a	7.15± 0.01b
J101CS	15901.97±4259.94a	365.00±30.64bc	20.87 ±0.52c	130.67±38.55a	7.11± 0.04b
J101BS	14519.37 ±3988.02a	385.00 ±2.36c	19.86 ±0.25c	105.00±0.00a	6.91± 0.05a
J101FL	15030.87 ±4613.85a	290.00±42.43±ab	18.05 ±0.40b	149.33±29.14a	6.90± 0.06a

CK: un-inoculation treatment. J101CS: application with clear supernatant, J101BS: inoculated with the bacteria, J101FL: inoculated with the mixture of supernatant and bacteria One-way analysis of variance was performed followed by Duncan's multiple range test at a significance level of $p<0.05$. Different letters indicate statistically significant differences.

Table 4 The α-diversity indices of bacterial and fungal communities in maize rhizosphere soil.

Inoculation	Bacterial community		Fungal community	
	Chao	Simpson	Chao	Simpson
CK	2,765.35± 9.07a	0.0086±0.0024a	647.85±51.30 a	0.27±0.17a
J101CS	2,685.36± 65.86a	0.0112±0.0047a	768.77±17.67b	0.05± 0.01b
J101BS	2,720.58± 10.36a	0.0085±0.0004a	743.18±19.82ab	0.05± 0.01b
J101FL	2,723.07± 39.56a	0.0077±0.0025a	804.30±44.27b	0.17± 0.03a

CK: non-inoculated, J101CS: application with clear supernatant, J101BS: inoculated with the bacteria, J101FL: inoculated with the mixture of supernatant and bacteria. One-way analysis of variance was performed followed by Duncan's multiple range test at a significance level of $p<0.05$. Different letters indicate statistically significant differences.
