# Supplementary material for: Pb-resistant Pantoea rwandensis promotes maize’s growth by altering Pb accumulation in biomass and soil Pb immobilization
Source: PLoS One. 2024 Oct 18;19(10):e0306392. doi: 10.1371/journal.pone.0306392 (PMC11488736; doi:10.1371/journal.pone.0306392)
Supplement: Attachment — Submitted filename: Comments for Manuscript Number PONE-D-23-42620.docx [file pone.0306392.s012.docx]

- “The line of argumentation should be improved by dividing the manuscript into clear sections with subheadings.”

This is an interesting, clear, concise, and well-written manuscript. The introduction is relevant and theory based. Sufficient information about the previous study findings is presented for readers to follow the present study rationale and procedures. The methods are generally appropriate. I enjoyed the manuscript and believe that it will be a relevant contribution to the field. It only needs to undergo a few minor changes before its publication. This paper has a potential to be accepted, but some important points have to be clarified basically in tables and figures. Below I have provided numerous remarks to correct the text.

- In several instances the authors develop a unique theoretical framework, and I believe that they should highlight their originality much more.
- The manuscript contains an elaborate literature, but definitions of the key concepts are needed in the introduction.
- The authors draw on extensive empirical evidence. I believe that they can put forward their arguments much more confidently.
- Throughout the manuscript, there are several minor language mistakes. Therefore, I recommend a certified round of language editing before the paper is published.
- It is very difficult to understand the figures and tables without legend. The authors are requested to put the proper titles along-with figures and tables. The information given under the title of supporting information (line no 672-674) is not adequate, in which only information is given regarding S1 only not for S2 and it is also not very clear.
- The titles given in every table (for example in table no 1, Change of biomass in maize) is not adequate and not given proper message
- In table 3, authors should write the abbreviations URE, ACP AP and AN
- No doubt that figure are impressive but authors should describe regarding alphabets like a,b,c etc. in the figure
- In figure 5, what is RDA1 and RDA 2
- Clear that what does Phylogenetic tree indicates, properly discuss in your text.
- Line No. 214 to 216; no need to write this is the fig 1 title. Title should be written with figures
- Line no 233 to 236, text should be written with table 1
- Check line 251-256 for title. Is it for fig 1 or 2.
- Line 264-269 write title with figure 3
- Line 286 to 290 write title with respective table
- Line no 292 activity of acid phosphatase is ok
- Line 301-305 write title with table
- Line 314-318 write title with table 4
- Line 334-336 write title with figure 4
- Line no 352-365 write text with fig 5 and try to reduce the text of title
- English Grammar Mistakes in Technical Manuals

Evaluation of the purposive approach to statutory interpretation

6: Finally I recommend that this paper be accepted after minor revision."1

2.

Top 10 English Grammar Mistakes in Technical Manuals

4:01

3.

Peer Sentences

2:30

4. DD

Writing a Critique Paper: 7 Easy Steps

12:11

5.

Evaluation of the Golden rule www.e-lawresources.co.uk

6:01

P
