# Supplementary material for: Pb-resistant Pantoea rwandensis promotes maize’s growth by altering Pb accumulation in biomass and soil Pb immobilization
Source: PLoS One. 2024 Oct 18;19(10):e0306392. doi: 10.1371/journal.pone.0306392 (PMC11488736; doi:10.1371/journal.pone.0306392)
Supplement: Attachment — Submitted filename: Response to Reviewers.docx [file pone.0306392.s013.docx]

PLOS ONE

Manuscript Number: PONE-D-23-42620

Title: Pb-resistant phosphate-solubilizing bacteria promote maize’s growth by altering Pb accumulation in biomass and soil Pb immobilization

Dear Editor and Reviewers,

Thank you very much for your letter and for the reviewers’ comments on our manuscript entitled ‘Pb-resistant phosphate-solubilizing bacteria promote maize’s growth by altering Pb accumulation in biomass and soil Pb immobilization’. Those comments are all valuable and very helpful for revising and improving our paper, as well as the important guiding significance to our research. We have substantially revised our manuscript and provided details in the current version. We have studied the comments carefully in the revised manuscript. We hope that this revision is acceptable and look forward to hearing from you soon.

With best wishes.

Sincerely Yours,

Xiuli Hou

Below, please find the comments in black, followed by our responses in red.

## Editor's Decision

Thank you for submitting your manuscript to PLOS ONE. After careful consideration, we feel that it has merit but does not fully meet PLOS ONE’s publication criteria as it currently stands. Therefore, we invite you to submit a revised version of the manuscript that addresses the points raised during the review process.

## Journal requirements:

When submitting your revision, we need you to address these additional requirements.

1. Please ensure that your manuscript meets PLOS ONE's style requirements, including those for file naming. The PLOS ONE style templates can be found at

<https://journals.plos.org/plosone/s/file?id=wjVg/PLOSOne_formatting_sample_main_body.pdf> and

<https://journals.plos.org/plosone/s/file?id=ba62/PLOSOne_formatting_sample_title_authors_affiliations.pdf>

**Response:** We are pleased to receive your letter. We have revised the format of the manuscript according to the requirements of the journal. If you have any questions about the review, you can contact us. We will actively cooperate with the work until the manuscript meets the requirements of the journal.

2. In your Methods section, please provide additional information regarding the permits you obtained for the work. Please ensure you have included the full name of the authority that approved the field site access and, if no permits were required, a brief statement explaining why.

**Response:** Thank you for raising the question. Our study site did not involve endangered or protected species, so no specific permissions were required for the location/activity. We have revised this part of the content in the manuscript, which you can find on line 113-114 of the manuscript.

3. We note that the grant information you provided in the ‘Funding Information’ and ‘Financial Disclosure’ sections do not match.

When you resubmit, please ensure that you provide the correct grant numbers for the awards you received for your study in the ‘Funding Information’ section.

**Response:** Thank you for your question. We will change the funding information while submitting the revised manuscript.

4. Thank you for stating the following in the Acknowledgments Section of your manuscript:

“This work is funded by National Natural Science Foundation of China (42167009), Joint Fund Project of Yunnan Provincial Universities (2018FH001-004), Scientific Research Fund Project of Yunnan Provincial Department of Education (2022Y712, 2023Y0874), The International Joint Innovation Team for Yunnan Plateau Lakes and Laurentian Great Lakes (funded by Yunnan Provincial Education Department), College Students' Innovation and Entrepreneurship project (202111393070).”

We note that you have provided funding information that is currently declared in your Funding Statement. However, funding information should not appear in the Acknowledgments section or other areas of your manuscript. We will only publish funding information present in the Funding Statement section of the online submission form.

Please remove any funding-related text from the manuscript and let us know how you would like to update your Funding Statement. Currently, your Funding Statement reads as follows:

“The author(s) received no specific funding for this work.”

Please include your amended statements within your cover letter; we will change the online submission form on your behalf.

**Response:** Thank you for your questions, we have deleted the funding information in the acknowledgment section of the manuscript. Our Acknowledgements section will be updated to the following: This project was supported the Department of College of Agronomy and Life Sciences, Kunming University, and Yunnan Collaborative Innovation Center for Plateau Lake Ecology and Environmental Health (funded by Yunnan Provincial Education Department). We also acknowledge the support of the College of Agronomy and Life Sciences for providing the greenhouse and laboratory facility for experimenting. We will write the revised statement to the cover letter and ask you to change the online submission form.

5. PLOS requires an ORCID iD for the corresponding author in Editorial Manager on papers submitted after December 6th, 2016. Please ensure that you have an ORCID iD and that it is validated in Editorial Manager. To do this, go to ‘Update my Information’ (in the upper left-hand corner of the main menu), and click on the Fetch/Validate link next to the ORCID field. This will take you to the ORCID site and allow you to create a new iD or authenticate a pre-existing iD in Editorial Manager. Please see the following video for instructions on linking an ORCID iD to your Editorial Manager account: <https://www.youtube.com/watch?v=_xcclfuvtxQ>.

6. Please amend the manuscript submission data (via Edit Submission) to include author Xuexiu Chang.

**Response:** We have modified the submission data.

7. Please include your tables as part of your main manuscript and remove the individual files. Please note that supplementary tables (should remain/ be uploaded) as separate "supporting information" files.

**Response:** Thank you for raising the question, we have revised that section of the content.

8. We notice that your supplementary figures are uploaded with the file type 'Figure'. Please amend the file type to 'Supporting Information'. Please ensure that each Supporting Information file has a legend listed in the manuscript after the references list.

**Response:** Thank you for raising the question, we have revised that section of the content.

**Additional Editor Comments:**

We have thoroughly reviewed your manuscript by reviewers and editors, and it requires major revision for publication. Please follow the comments and resubmit your revised manuscript with the reviewers' responses.

Reviewer #1: This study examines the efficacy of Pantoea rwandensis in reducing the availability and plant uptake of lead (Pb) in Pb-contaminated soil. The results of the study indicate that P. rwandensis solubilized tricalcium phosphate by secreting various organic acids and also improved maize growth and enhanced the diversity of fungi and bacteria in Pb-contaminated soils. From the results, it was concluded that the P-solubilizing P. rwandensis affected the transformation of Pb fractions in the soil through alterations in the functions of the soil bacteria thereby decreasing Pb accumulation in maize and improving its growth. Although the results are interesting, there are several concerns as mentioned below that need attention. As the changes are numerous to list here, I marked them directly in the annotated manuscript.

1. The manuscript should be language edited to improve readability. The sentences are wordy and incomplete making reading and understanding difficult. I would suggest the authors seek the help of a colleague with good proficiency in English to language edit the manuscript.

**Response:** Thank you for raising the issue. I apologize for any inconvenience caused by grammatical errors, lengthy sentences, and incompleteness in the manuscript. We have thoroughly revised the manuscript with professional language editing to correct grammar, style, and typos.

2. There are already studies where species in Pantoea are reported to possess nutrient solubilizing, plant growth, and heavy metal tolerance (e.g., <https://doi.org/10.1111/14622920.14946>; <https://doi.org/10.3390/microorganisms8020153>; <https://doi.org/10.1007/s11274-019-2744-4>; <https://doi.org/10.3390/microorganisms9081661>).

Unfortunately, previous studies available on the plant-growth-promoting activities of Pantoea are not adequately summarized in the introduction.

**Response:** Thank you for your valuable suggestions. We have revised the introduction in our manuscript and added a summary of the characteristics of species within Pantoea, including nutrient solubilization, plant growth promotion, and heavy metal tolerance, to make the content and structure of the article clearer. You can find this in line 72-79 of the manuscript.

3. The study is confounded by methodological shortfalls. For instance, the phosphatic source used for the determination of phosphate solubilization is not correct. Tricalcium phosphate is a weak phosphatic source that can be solubilized by any acid-producing bacteria including Escherichia coli. To determine true phosphate-solubilization, a harder-to-solubilize phosphatic source like aluminium phosphate or iron phosphate has to be used. If the bacterial isolate can solubilize these hard-to-solubilize phosphatic sources then it will invariably solubilize tricalcium phosphate. For more details, see <https://doi.org/10.1007/s00374-012-0756-4>.

**Response:** I would like to express my gratitude for your careful review of our research and for providing valuable suggestions. You have raised a significant concern regarding the use of tricalcium phosphate as a phosphorus source to assess the phosphate solubilizing abilities of bacterial isolates. Indeed, as you mentioned, tricalcium phosphate is considered a weaker and more soluble form of inorganic phosphorus that can be utilized by various acid-producing microorganisms. Therefore, it may not sufficiently differentiate microbes with high efficiency in dissolving insoluble inorganic phosphate compounds under certain conditions. Following your feedback, we agree that using more insoluble sources such as aluminum phosphate or iron phosphate would provide a more accurate evaluation of the true solubilization capabilities of bacterial isolates. In future work, we plan to adopt this approach and conduct corresponding experiments to validate our current results and explore other potential efficacies. Although tricalcium phosphate has lower solubility compared to other forms of phosphorus sources, its solubility increases slightly under mildly acidic conditions (pH just below 7). This could simulate the release process of inorganic phosphorus under slightly acidic conditions found in natural environments such as soil. Given its extensive historical documentation in literature records, tricalcium phosphate is often used as a source of phosphorus for determining the solubilizing ability of acid-producing microorganisms. We believe that there are merits to using tricalcium phosphate as a source for exploring bacterial phosphorus-solubilizing capabilities.

For relevant information please refer to these articles:

<https://www.nature.com/articles/s41598-021-88635-4>;

https//www.doi.[10.1007/s00284-023-03408-8](https://www.x-mol.com/paperRedirect/1681840166319931392);

<https://doi.org/10.1186/s12866-022-02715-7>.

4. Similarly, Salkowski’s reagent can react with a wide range of indole compounds (e.g., indole-acetamide, indole-3-pyruvic acid) to produce colour and is not specific to indole acetic acid. To be more precise, a more reliable technique using HPLC or LC-MS should be used for determining indole acetic acid production (consult <https://doi.org/10.3389/fchem.2018.00265>; <https://doi.org/10.1128/aem.61.2.793-796.1995>).

**Response:** Thank you very much for your detailed review and valuable suggestions. We acknowledge that the Salkowski reagent may cross-react with other indole compounds when detecting indole-3-acetic acid (IAA), thereby affecting specificity. We fully agree with your point that using high-performance liquid chromatography (HPLC) or liquid chromatography-mass spectrometry (LC-MS) for quantifying IAA would provide more accurate and reliable results. Our experimental method was established by referencing the literature of several researchers, as indicated by sources such as (<https://doi.org/10.1186/s12866-022-02715-7>; <https://doi.org/10.1186/s12866-022-02715-7>). As you mentioned, this could lead to biases in our experimental outcomes. Our sincerely apologize for the lack of rigor in our work and will consider employing advanced techniques such as HPLC or LC-MS in future studies to obtain more precise data regarding IAA production levels.

5. The methods section requires a rewriting by incorporating missing information. Important information like the plant species from which the soil samples were collected, tailing soil characteristics, the depth and quantity of soil collected, source of maize seeds, experimental design (completely randomized block design, etc.), etc., are missing.

**Response:** Thank you for your meticulous review and valuable comments on our research paper. You have pointed out the issue of missing key information in the Methods section, which is crucial for the replication and understanding of experimental results. We sincerely apologize for omitting details such as plant species during soil sample collection, characteristics of tailings soil, sampling depth and quantity, as well as the source of maize seeds—oversights we admit were not adequately addressed in our writing process. To adhere to scientific sharing principles and enhance reader trust, we will thoroughly supplement all necessary data and methodological details in the revised manuscript. Thank you for your precise and constructive critique; it has undoubtedly helped us improve and strengthen the quality of our paper.

6. as any standard method used for collecting the rhizosphere soil? If not state how rhizosphere soil was differentiated from the surrounding bulk soil.

**Response:** Thank you for your valuable feedback. We recognize that there is no widely accepted and uniformly implemented standard method for rhizosphere soil collection. The method we adopted is designed to best meet the objectives of our study while considering the limitations of field conditions. In this study, we have clearly defined in the methods section the distinction between rhizosphere soil and bulk soil, and based on recommended practices from published literature, we made modifications to suit our experimental needs. Briefly described, after carefully removing surrounding loose soil, we collected the soil closely associated with plant roots for bacterial isolation screening and microbial diversity analysis; the remaining removed loose soil was considered non-rhizosphere soil. Additionally, all other soils used for chemical analyses were non-rhizosphere soils, which has been corrected in our manuscript.

7. Explain how different concentrations of Pb were fixed to test the tolerance of the bacterium to the heavy metal.

**Response:** Thank you for your question. In conducting the lead resistance experiment on bacterial strains, we followed the procedures outlined in reference literature as follows:

1. Stock Solution Preparation: Calculate and weigh out an adequate amount of Pb(NO_3_)_2_ powder to prepare a high-concentration stock solution. For instance, you might start by preparing a 1000 mg·L^-1^ stock solution. Dissolve the weighed Pb(NO_3_)_2_ powder in deionized water or another appropriate solvent, and use magnetic stirring to ensure complete dissolution.
2. To prepare working solutions with different concentrations (750, 500, and 250 mg·L^-1^) by dilution from a stock solution that has already been prepared at 1000 mg·L^-1^. For example, to obtain a 500 mg·L^-1^ working solution, take half the volume of the stock solution and mix it with an equal volume of deionized water.
3. Add Pb(NO_3_)_2_ to the fermentation liquid: For each group with a different lead nitrate concentration to be tested, add a pre-calculated volume of Pb(NO_3_)_2_ working solution that has been diluted to the respective target concentrations (250, 500, 750, or 1000 mg·L^-1^).

Note: The control group should receive an equivalent volume of deionized water without Pb(NO_3_)_2_ or another suitable solvent for this type of experiment.

8. The criteria used for the selection of the isolate J101 for further studies are missing.

**Response:** We sincerely appreciate your meticulous review of our work and the valuable comments provided. Regarding your point about the lack of criteria for selecting isolate J101 for further study, we have carefully considered your suggestion and supplemented it in the Materials and Methods section. You can find this addition in line 127-136 of the manuscript.

9. Parametric analysis like ANOVA requires normal distribution of data. Therefore it is important to check the homogeneity of the data. If the data fails to satisfy normality, then it should be transformed. Non-parametric analysis should be used if the data fails to satisfy normality even after transformation. Moreover, although Post Hoc analysis (Duncan’s multiple range test) was used mean separations are not presented for those in Figure 1.

**Response:** Thank you for your valuable feedback. In Figure 1, we did not perform a one-way ANOVA on the data. Figure 1A shows the relationship between the content of soluble phosphorus in the culture fluid of strains and changes in pH value and culture time. Figure 1B illustrates the variation in OD_600_ values of strains cultured with different concentrations of lead ions. Figure 1C presents the amount of organic acids and IAA secreted by strains. As can be seen, the data in Figure 1 merely compares their numerical values without performing a one-way ANOVA analysis between groups. The reason why mean separations are not presented in some parts of Figure 1 is that within a group, there are three replicates whose values are quite close to each other; this results in smaller errors among these values, leading to smaller standard errors which make mean separation less visible in certain datasets when graphically represented.

10. The total number of PSB isolates in the tailing soil samples is missing in the results. Remove the data that are already there in the tables and figures from the results.

**Response:** Thank you for your valuable comments. We regret the omission of the total number of PSB isolates from tailings soil samples in our results. We have now included the total count of PSB isolates from tailings soil samples in the revised manuscript, which can be found on line 247. Additionally, we have removed data that was already presented in tables and figures from the results section.

11. As the treatments are compared with a control Dunnett Post Hoc analysis would be more appropriate than DMRT.

**Response:** Thank you for your meticulous review and valuable suggestions regarding our research work. Regarding the use of Dunnett's post hoc analysis instead of DMRT to compare the effects between treatment groups and control, we have given this careful consideration. Given that the Dunnett test is specifically designed to compare multiple treatment groups with a single control group and can effectively control the Type I error rate, we acknowledge its suitability for certain comparisons. However, upon further evaluation, we believe that in our study not only did we compare treatment groups with the control group but also made comparisons among treatment groups themselves. As stated in the introduction section, our study aims to explore the effects of phosphate-solubilizing bacteria (PSB) and their metabolic products as well as their combined application on soil chemical properties, lead speciation, growth promotion in maize plants, and soil microbial community structure. In addition, in our discussion section, we analyzed differences in experimental results formed between three different treatments; hence our focus was on comparing effects from culture filtrate alone versus bacterial isolates versus their combined application. Therefore, considering these aspects of inter-treatment comparison alongside those against controls within our analyses led us to choose DMRT for statistical analysis purposes.

12. Many parts of the discussion are repetitions of the results. Some of the results are not adequately discussed. For instance, the combined application of culture filtrate and the bacterial isolate failed to improve maize growth compared to their inoculation. Nevertheless, the reasons for this decreased intensity in plant growth are not adequately discussed.

**Response:** Thank you for your valuable comments regarding the issues of repetitive results and some outcomes not being fully discussed in various parts of our discussion. We have conducted a thorough review of the manuscript and provided an extensive discussion of the results in the discussion section.

Other comments:-

13. Line 44–45: Avoid using words that are already there in the title as keywords.

**Response:** Thank you for your valuable suggestions. We have revised the keywords accordingly. In the manuscript, this can be found on line 42-43.

**For your mark in the manuscript, we made the following response:**

- **Line 31-32 not clear.**

**Response:** Thank you for raising the question. We have revised this part of the content in the manuscript, which you can find on line 29-31 of the manuscript.

- **Line 32'Culture filtrate' is more appropriate for than clear supernatant. Change it throughout the manuscript.**

**Response:** Thank you for your suggestion. We have replaced "clear supernatant" with "Culture filtrate" throughout the manuscript.

- **Line 39-40 How could this be inferred without studying?**

**Response:** Thank you for raising the question. In the original text, this section was based on existing literature where similar research work showed that inoculation with Pantoea strains increased the relative abundance of heavy metal-tolerant microbes in soil. Therefore, we made an inference that Pantoea could achieve the same effect in our study. Clearly, this is not reasonable and we have removed this content from our manuscript.

- **Line 93 Provide more details on the tailing site. What are the environmental conditions and vegetation on the tailing site?**

**Response:** Thank you for your suggestion. We have updated the information in our manuscript, and you can find this section on line 108-113 of the manuscript.

- **Line 95 Does this mean healthy plants?**

**Response:** Thank you for raising the question. We have made corrections in the manuscript, and what is described here refers to healthy plants.

- **Line 97 What strain? Bacteria, fungi, etc.**

**Response:** Thank you for raising the question; this strain is a bacterium, and we have made the correction in our manuscript.

- **Line 98-101 There is no need to detail the standard serial dilution technique used.**

**Response:** Thank you for raising the question, we have revised that section of the content.

- **Line 102-103 There is no need to provide the composition of the medium unless there is some modification.**

**Response:** Thank you for raising the question; we have revised that section of the content.

- **Line 110 How was this determined?**

**Response:** Thank you for your question. Determining the concentration of a bacterial suspension typically requires dilution and plate count methods. Here are the general steps:

1. Prepare an original suspension: First, take an appropriate amount from a pure culture of bacteria and make an original suspension with sterile saline or another suitable buffer solution.
2. Perform serial dilutions: Dilute the original suspension at certain ratios (e.g., 1:10, 1:100, etc.) to obtain samples within a range that can be accurately counted.
3. Plate counting method: Inoculate a fixed volume (such as 0.1 mL or 1 mL) from each level of dilution onto solid media plates and then incubate under constant temperature conditions until visible individual colonies form.
4. Calculate CFU/mL: After incubation, select plates with about 30-300 colonies (considered to have minimal error and easy to count), count them, and calculate the number of viable cells per milliliter in the original suspension (CFU/mL).
5. CFU/mL = (Average colony count / Inoculation volume) × Dilution factor
6. Adjust final concentration: If initial testing shows that you've achieved your target density, no further action is needed; if not, you may need to adjust your original suspension to achieve the correct density by adding more untreated filtrate or further diluting it.
7. Verification and repeatability check: To ensure accuracy, it's recommended to repeat this process at least three times and use statistical methods such as calculating mean values and standard deviations to assess data quality and reliability.
8. In some cases, special equipment like flow cytometers or spectrophotometry can also be used for rapid estimation of microbial numbers but usually require calibration against traditional methods.

- **Line 116-117 How PH?**

**Response:** Thank you for your question. The pH value mentioned is the pH of the culture medium, which is reflected in Figure 1 of the results analysis.

- **Line 130-131 Confusing. What is the need for diluting the medium with sterile water? Control should be similar to culture except the presence of bacteria.**

**Response:** Thank you for your question. Prior to bacterial inoculation, we dilute the culture medium with sterile water in order to standardize experimental conditions. The purpose of this is to ensure comparability between different experiments or when the same experiment is repeated. It's necessary to have a uniform standard because it's not possible to precisely control the number of bacteria during each cultivation due to variations in cultivation time and inoculum size. Therefore, using a fixed concentration of bacterial suspension before starting new experiments can make the results more stable and reliable.

- **Line 133 This sentence is incomplete.**

**Response:** Thank you for raising the question, we have revised that section of the content.

- **Line 145 Provide the geographical coordinates.**

**Response:** Thank you for your suggestion. We have updated the information in our manuscript, and you can find this section on line 179-180 of the manuscript.

- **Line 147-148 How were these determined.**

**Response:** Thank you for your suggestion. We have updated the information in our manuscript, and you can find this section on line 213-214 of the manuscript.

- **Line 155 Express centrifugal force in 'g' units.**

**Response:** Thank you for raising the question, we have revised that section of the content.

- **Line 166-167 What was the light intensity and relative humidity in the greenhouse?**

**Response:** Thank you for your suggestion. We have updated the information in our manuscript, and you can find this section on line 198 of the manuscript.

- **Line 169 How much soil was collected?**

**Response:** Thank you for your suggestion. We have updated the information in our manuscript, and you can find this section on line 209 of the manuscript.

- **Line 195 Fermentation is a process.**

**Response:** Thank you for raising the question, we have revised that section of the content.

- **Line 199 This needs mention in the methods.**

**Response:** Thank you for your suggestion. We have updated the information in our manuscript, and you can find this section on line 145-146 of the manuscript.

- **Line 203 Is it the growth?**

**Response:** Thank you for raising the question, we have revised that section of the content. It is growth.

- **Line 208 Are you sure that only these organic acids were secreted by the bacterial isolate?**

**Response:** When we use liquid chromatography to analyze the organic acid content secreted by strains, oxalic acid, tartaric acid, lactic acid, and acetic acid exhibit higher absorption peaks in the spectrum and are clearly visible. However, for other organic acids, their retention times in the spectrum are shorter and their peak values are smaller or even undetectable. Therefore, these four organic acids are present in the highest amounts and represent the main types of organic acids secreted by the strain.

- **Line 210-211 What is the purpose of inferring the results when there is a separate discussion section.**

**Response:** Thank you for raising the question, we have revised that section of the content.

- **Line 218-211 Was this the only PSB isolate present in the tailing soil.**

**Response:** Thank you for your question. This is not the only strain isolated from tailings soil, but subsequent analysis and identification were conducted only on this particular strain which showed promising results. The content presented here merely expresses the identification results of strain J101.

- **Line 225 Replace above ground with shoot throughout the manuscript.**

**Response:** Thank you for your question; we have revised the entire manuscript accordingly.

- **Line 369 Repeat of the results.**

**Response:** Thank you for your question. We have revised the entire manuscript, removing all repetitive results sections that appeared in the discussions.

- **Line 377-378 How could you compare a rhizosphere bacteria with nodulating N2-fixing bacteria?**

**Response:** Thank you for your question. Although this strain is a nitrogen-fixing bacterium, literature review reveals that it also possesses the same phosphate-solubilizing function, and its mechanism of solubilizing phosphate is similarly through the secretion of organic acids. Nitrogen fixation is just one of the functions of this strain. In addition, studies have shown that different phosphate-solubilizing bacterial species share many common functions such as secreting organic acids, producing indole-3-acetic acid (IAA), fixing nitrogen, solubilizing phosphates and potassium, and producing siderophores.

- **Line 395 This is not a scientific term.**

**Response:** Thank you for pointing out the issue; we have now corrected the name to the proper Latin form.

- **Line 437 End the sentence after the figure citation.**

**Response:** Thank you for raising the issue; we have now corrected the format in the manuscript.

- **Table1 This is already there in the methods; Should it be read row wise or column wise?**

**Response:** Thank you for your question. We have made the corresponding annotations in the table explanation of our manuscript.

Reviewer #2: The manuscript entitled “Pb-resistant phosphate-solubilizing bacteria promote maize’s growth by altering Pb accumulation in biomass and soil Pb immobilization”. (PONE-D-23-42620)
The authors contribute to evaluate that application of phosphate solubilizing bacteria (PSB) to reduce the phytoavailability of Pb uptake by maize plant. The main results were inoculation with P. rwandensis could promote the transformation of Pb fractions, and affected the soil bacteria community, and finally promoted the growth of maize. The work is helpful in usage of PSB strain. However, there still several major issues should be revised. Moreover, the language should be further improved.

1. Major concerns:The goal of this work should be clearer. I think more information should be summarized to present the novelty of this work. What is the great difference of this strain from previous studies, and the new finding in this work should be provided. Why is the maize plant used in this study? The reasons should be mentioned in the Introduction section.

**Response:** Thank you for your valuable suggestions. We have revised the manuscript accordingly and made corrections in the revised version. In response to your comments regarding highlighting the novel findings of our study and explaining why we used corn plants, we have addressed these issues in the text of the manuscript.

2. Minor concerns: The authors should revise the text. Lots of minor revisions should be paid more attention. For instance, Line 64 PSB when the first time to describe, it should be give full name.

**Response:** Thank you for your valuable comments. We have revised the manuscript so that "PSB" is now presented in its full form upon its first occurrence. You can find this change on line 63 of the manuscript.

3. Line 68 to 70, should be revised.

**Response:** Thank you for your suggestion. We have updated the information in our manuscript, and you can find this section on line 69-71 of the manuscript.

4. Line 89 to 90, should be revised.

**Response:** Thank you for your suggestion. We have updated the information in our manuscript.

5. Line 98 to 100, should be revised.

**Response:** Thank you for your suggestion. We have updated the information in our manuscript, and you can find this section on line 121-123 of the manuscript.

6. Line 153 strain J101.

**Response:** Thank you for your suggestion. We have updated the information in our manuscript.

7. Line 168 should be revised.

**Response:** Thank you for your suggestion. We have updated the information in our manuscript.

8. Line 189 to 191 The software version and full website should be revised.

**Response:** Thank you for your suggestion. We have updated the information in our manuscript, and you can find this section on line 241-244 of the manuscript.

9. Line 208 and 212 please check and revise.

**Response:** Thank you for your suggestion. We have updated the information in our manuscript, and you can find this section on line 261-263 of the manuscript.

10. Line 227-232 This section should be revised, what is your main results.

**Response:** Thank you for your suggestion. We have updated the information in our manuscript, and you can find this section on line 277-281 of the manuscript.

11. Line 247 to 248 MDA please use full name in figure caption.

**Response:** Thank you for your valuable feedback. We have revised the manuscript accordingly and included explanations in the figure captions.

12. Line 369 please revise.

**Response:** Thank you for your suggestion. We have updated the information in our manuscript, and you can find this section on line 401 of the manuscript.

13. Line 378-379 please revise.

**Response:** Thank you for your suggestion. We have updated the information in our manuscript, and you can find this section on line 408-410 of the manuscript.

14. Line 393 please revise. Please use the author names, not reference number.

**Response:** Thank you for your suggestion. We have updated the information in our manuscript, and you can find this section on line 422 of the manuscript.

15. Line 401 please revise.

**Response:** Thank you for your suggestion. We have updated the information in our manuscript, and you can find this section on line 431 of the manuscript.

16. Line 439 please revise.

**Response:** Thank you for your suggestion. We have updated the information in our manuscript, and you can find this section on line 476-477 of the manuscript.

17. Line 469, 473, 475, 477, 479, this section should be completely revised.

**Response:** Thank you for your suggestion. We have updated the information in our manuscript, and you can find this section on line 526-542 of the manuscript.

18. Figure 1 please revise. Figure 1 and Figure S1 and S2 can be merged into one figure.

**Response:** Thank you for your valuable suggestions. We have revised Figure 1 and, following your advice, combined Figures 1, S1, and S2 into a single figure as shown in Figure 1.

19. Although I can not provide each minor revision, the authors should pay more attention to your text, especially the language. The goal is to make your work readable and useful to readers.

**Response:** Thank you for your valuable feedback. I apologize for any inconvenience caused by the grammatical errors and other issues in the manuscript. We have now thoroughly revised the manuscript with professional language editing, correcting grammar, style, and typos to hopefully provide you with a more enjoyable reading experience.

Reviewer #3: This is an interesting, clear, concise, and well-written manuscript. The introduction is relevant and theory based. Sufficient information about the previous study findings is presented for readers to follow the present study rationale and procedures. The methods are generally appropriate. I enjoyed the manuscript and believe that it will be a relevant contribution to the field. It only needs to undergo a few minor changes before its publication. This paper has a potential to be accepted, but some important points have to be clarified basically in tables and figures. Below I have provided numerous remarks to correct the text.

1. In several instances the authors develop a unique theoretical framework, and I believe that they should highlight their originality much more.

**Response:** Thank you for your valuable feedback and high appreciation of our article. We have made revisions in response to the issues you raised. Following your suggestions, we have modified the introduction section to emphasize the originality and novelty of our research content.

2. The manuscript contains an elaborate literature, but definitions of the key concepts are needed in the introduction.

**Response:** Thank you for your valuable comments, we have defined the key concepts in the introduction under your suggestion.

3. The authors draw on extensive empirical evidence. I believe that they can put forward their arguments much more confidently.

**Response:** Thank you for your valuable comments. We have expressed our arguments more in the revised manuscript under your suggestions. You can see some of our new ideas in the discussion section of the manuscript.

4. Throughout the manuscript, there are several minor language mistakes. Therefore, I recommend a certified round of language editing before the paper is published.

**Response:** Thank you for your valuable feedback. I apologize for any inconvenience caused by the grammatical errors and other issues in the manuscript. We have now thoroughly revised the manuscript with professional language editing, correcting grammar, style, and typos to hopefully provide you with a more enjoyable reading experience.

5. It is very difficult to understand the figures and tables without legend. The authors are requested to put the proper titles along-with figures and tables. The information given under the title of supporting information (line no 672-674) is not adequate, in which only information is given regarding S1 only not for S2 and it is also not very clear.

**Response:** Thank you for your valuable feedback. We sincerely apologize for any inconvenience caused by inadvertently separating the figure captions and table titles from their corresponding figures and tables in our manuscript. We have now adjusted the placement of the icons within the manuscript, ensuring that appropriate titles are placed together with their respective figures and tables to enhance your reading experience. We have also removed content from the supporting information section and made modifications to Figure 1. Figures 1, S1, and S2 have been combined into a single figure for your convenience. You can find all relevant details in Figure 1.

6. The titles given in every table (for example in table no 1, Change of biomass in maize) is not adequate and not given proper message.

**Response:** Thank you for your valuable comments, we have comprehensively reviewed the chart titles that appear in the manuscript and revised them.

7. In table 3, authors should write the abbreviations URE, ACP AP and AN

**Response:** Thank you for your valuable comments, we have modified Table 3 and wrote the full name of URE, ACP AP and AN in the table description.

8. No doubt that figure are impressive but authors should describe regarding alphabets like a,b,c etc. in the figure.

**Response:** Thank you for your valuable comments. We have described the letters a, b, c in the manuscript, which you can see in the illustration.

9. In figure 5, what is RDA1 and RDA 2

**Response:** Redundancy Analysis (RDA) is a multivariate statistical method that combines aspects of Principal Component Analysis (PCA) and Canonical Correspondence Analysis (CCA). It is used to explore the relationship between environmental factors and species composition by extracting linear response models to explain variation within species data, linking this variation to a set of explanatory environmental variables. In an RDA plot:

- RDA1 represents the first principal axis or the first redundancy axis. It captures the variance in the species dataset that can be explained by environmental variables to the greatest extent. In other words, RDA1 indicates the most significant trend or gradient associated with environmental factors affecting species composition among all possible linear trends or gradients.

- RDA2 represents the second principal axis or second redundancy axis. It describes the next most important source of variance driven by environmental factors that can be detected in addition to what is explained by RDA1.

The length of each arrow reflects how much a particular environmental factor contributes to the overall structure of data; arrow direction shows how different factors interact together on sample sites and/or species; while distances between points visualize levels of similarity between different sample sites and/or species.

10. Clear that what does Phylogenetic tree indicates, properly discuss in your text.

**Response:** Thank you for your valuable comments, we re-explained the phylogenetic tree in the material method and result analysis, but the phylogenetic tree was only used as a strong evidence in the identification of strains, so when we learned through the phylogenetic tree what kind of strains were identified, we got the results we wanted, so we did not explain this part too much in the discussion.

11. Line No. 214 to 216; no need to write this is the fig 1 title. Title should be written with figures

**Response:** Thank you for your suggestion. We have updated the information in our manuscript, and you can find this section on line 272-275 of the manuscript.

12. Line no 233 to 236, text should be written with table 1

**Response:** Thank you for your suggestion. We have updated the information in our manuscript, and you can find this section on line 282 of the manuscript.

13. Check line 251-256 for title. Is it for fig 1 or 2.

**Response:** Thank you for your suggestion. We have updated the information in our manuscript, and you can find this section on line 300-302 of the manuscript.

14. Line 264-269 write title with figure 3

**Response:** Thank you for your suggestion. We have updated the information in our manuscript, and you can find this section on line 311-312 of the manuscript.

15. Line 286 to 290 write title with respective table

**Response:** Thank you for your suggestion. We have updated the information in our manuscript, and you can find this section on line 329 of the manuscript.

16. Line no 292 activity of acid phosphatase is ok

**Response:** Thank you for your suggestion. We have updated the information in our manuscript, and you can find this section on line 332 of the manuscript.

17. Line 301-305 write title with table

**Response:** Thank you for your suggestion. We have updated the information in our manuscript, and you can find this section on line 343 of the manuscript.

18. Line 314-318 write title with table 4

**Response:** Thank you for your suggestion. We have updated the information in our manuscript, and you can find this section on line 354 of the manuscript.

19. Line 334-336 write title with figure 4

**Response:** Thank you for your suggestion. We have updated the information in our manuscript, and you can find this section on line 373-376 of the manuscript.

20. Line no 352-365 write text with fig 5 and try to reduce the text of title.

**Response:** Thank you for your suggestion. We have updated the information in our manuscript, and you can find this section on line 393-396 of the manuscript.

21. Finally I recommend that this paper be accepted after minor revision.

**Response:** Thank you for your valuable comments, which will inspire us to constantly improve the content of the article and improve the quality of the article.
