# Supplementary material for: Pb-resistant Pantoea rwandensis promotes maize’s growth by altering Pb accumulation in biomass and soil Pb immobilization
Source: PLoS One. 2024 Oct 18;19(10):e0306392. doi: 10.1371/journal.pone.0306392 (PMC11488736; doi:10.1371/journal.pone.0306392)
Supplement: Attachment — Submitted filename: Decision for Manuscript No PONE-D-23-42620.docx [file pone.0306392.s014.docx]

Dear sir/madam

Thanking your for giving opportunity again for re-evaluating the R paper entitled Pb-resistant *Pantoea rwandensis* promotes maize's growth by altering Pb accumulation in biomass and soil Pb immobilization.

Now the authors have made all the corrections given by me and modify the manuscript to emphasize the originality of the research. They have thoroughly revised the manuscript by mentioning the appropriate titles with their respective figures and describing specific alphabets with complete information of analysis, by adjusting the placement of the icons within the manuscript and correcting grammar and professional language editing. Now paper is in the position for publication.

Finally I recommend this manuscript for final publication.

Regards

Dr Deepti Shankhdhar
